# Delivering the CRISPR/Cas9 system for engineering gene therapies: Recent cargo and delivery approaches for clinical translation

**DOI:** 10.3389/fbioe.2022.973326

**Published:** 2022-09-26

**Authors:** Ruth A. Foley, Ruby A. Sims, Emily C. Duggan, Jessica K. Olmedo, Rachel Ma, Steven J. Jonas

**Affiliations:** ^1^ Department of Pediatrics, David Geffen School of Medicine, University of California, Los Angeles, CA, United States; ^2^ Department of Bioengineering, University of California, Los Angeles, CA, United States; ^3^ California NanoSystems Institute, University of California, Los Angeles, CA, United States; ^4^ Eli and Edythe Broad Center of Regenerative Medicine and Stem Cell Research, University of California, Los Angeles, CA, United States

**Keywords:** gene therapy, CRISPR/Cas9, genome editing, intracellular delivery, nano carriers

## Abstract

Clustered Regularly Interspaced Short Palindromic Repeats associated protein 9 (CRISPR/Cas9) has transformed our ability to edit the human genome selectively. This technology has quickly become the most standardized and reproducible gene editing tool available. Catalyzing rapid advances in biomedical research and genetic engineering, the CRISPR/Cas9 system offers great potential to provide diagnostic and therapeutic options for the prevention and treatment of currently incurable single-gene and more complex human diseases. However, significant barriers to the clinical application of CRISPR/Cas9 remain. While *in vitro, ex vivo,* and *in vivo* gene editing has been demonstrated extensively in a laboratory setting, the translation to clinical studies is currently limited by shortfalls in the precision, scalability, and efficiency of delivering CRISPR/Cas9-associated reagents to their intended therapeutic targets. To overcome these challenges, recent advancements manipulate both the delivery cargo and vehicles used to transport CRISPR/Cas9 reagents. With the choice of cargo informing the delivery vehicle, both must be optimized for precision and efficiency. This review aims to summarize current bioengineering approaches to applying CRISPR/Cas9 gene editing tools towards the development of emerging cellular therapeutics, focusing on its two main engineerable components: the delivery vehicle and the gene editing cargo it carries. The contemporary barriers to biomedical applications are discussed within the context of key considerations to be made in the optimization of CRISPR/Cas9 for widespread clinical translation.

## Introduction

The discovery of efficient genome-editing tools such as the Clustered Regularly Interspaced Short Palindromic Repeats-associated protein 9 (CRISPR/Cas9) nuclease system has revolutionized our ability to manipulate the human genome. The selective editing of targeted DNA sequences enabled by these genetic engineering tools facilitates the permanent correction of genomic mutations, paving the way for new potential treatments for many genetic diseases. Based on exploitation of the natural immune system of *Streptococcus pyogenes* and an understanding of the fundamental structural function of RNA enzymes present in bacteria, the coupling of CRISPR and Cas9 to create a powerful gene editing tool earned colleagues Emmanuelle Charpentier and Jennifer Doudna the 2020 Nobel Prize in Chemistry. The path to widespread adoption of CRISPR/Cas9 as a genome editing tool began with an investigation into the mechanism of adaptive bacterial immunity. Initial findings, published in 2012, showed that programmed CRISPR/Cas9 and guide RNA could effectively cut viral DNA at sequence-specific sites (Jinek et al., 2012). Elucidation of the role of RNA in bacterial immunity against viral modifications to genomic DNA led to the discovery of two types of RNA that guide Cas9 to the DNA cut site (Jinek et al., 2013), and allowed for the simplification of this natural system to require just two components: Cas9 and a programmable single guide RNA sequence (sgRNA) (Cornu et al., 2017).

The CRISPR/Cas9 system utilizes the programmable sgRNA to locate and bind to specific regions of the genome, where the Cas9 nuclease induces double-strand breaks (DSBs) at the target locations indicated by the guide sequence. The correction of defective endogenous genes can then occur either by removing specified regions of the target gene or by inserting an exogenous strand of DNA, dependent upon the DSB repair mechanism. Knockouts can occur if the DSB is repaired by non-homologous end joining (NHEJ) using protein factor re-ligation, while homology-directed repair (HDR) uses a homologous repair template to repair the DSB precisely, introducing a donor DNA template sequence of choice (Komor et al., 2016; Cullot et al., 2019; Wei et al., 2020; Yip, 2020; Sharma et al., 2021). Given that CRISPR/Cas9 allows for targeted DNA editing and only requires the relatively simple design of a guide RNA, it remains the most cost effective, standardized, and reproducible gene editing tool currently available (Bhattacharya et al., 2015; Yang et al., 2017). The emergence of this robust method for coordinating the manipulation of the genome has not only increased mechanistic understanding of intrinsic DNA repair processes, but is accelerating the development of treatments for genetic diseases *via* gene silencing, insertion, or site-specific correction.

Potentially curative gene editing efficiencies in the lab, such as CRISPR/Cas9-mediated editing to achieve over 20% efficiencies in human hematopoietic stem cell populations using a Cas9 ribonucleoprotein (RNP) complexed to a single-stranded DNA oligonucleotide donor (ssODN) (Magis et al., 2022), have paved the way for the first clinical trials that apply CRISPR based therapies. Recently announced phase I and II clinical trials that leverage CRISPR/Cas9-based strategies to treat transfusion-dependent β-thalassemia (NCT03655678), sickle cell disease (NCT03745287) (Frangoul et al., 2021), transthyretin amyloidosis (NCT04601051) (Gillmore et al., 2021) and Leber congenital amaurosis 10 (NCT03872479) (Mullard, 2019) demonstrate the potential to treat monogenetic disorders with a single, consistent base pair mutation. However, the clinical translation of CRISPR-based therapies becomes increasingly more complex as the number and heterogeneity of mutations increases. One solution to this issue involves the integration of a normal copy of the associated complementary DNA (cDNA) upstream of the known, disease-causing mutations. For example, Kuo et al. (2018) demonstrated the site-specific incorporation of a human codon-divergent CD40L cDNA at the 5′ UTR of the gene in both primary patient T lymphocytes and human CD34^+^ hematopoietic stem cells, resulting in expression of the therapeutic gene and effectively muting all downstream, disease-causing mutations (Kuo et al., 2018). Further studies into the mechanism of CRISPR/Cas9 function, including the kinetics of DNA recognition, the binding mechanism of the Cas9 protein that enables it to ‘read’ DNA (Redding et al., 2015), and the kinetics of Cas9 DNA interrogation (Cofsky et al., 2022), continue to be conducted with the aim of improving clinical translatability. However, the secrets behind the incredible efficiency of this protein interrogation system, the impact of target search speed, and the natural diversity in limiting this efficiency all remain poorly understood. Elucidating these phenomena may enable the manufacture of faster search speeds and increased CRISPR/Cas9 efficiency in future clinical settings.

Other CRISPR/Cas9-based technologies such as base editing (BE) and prime editing (PE) are some of the newest evolutions of gene editing methods that can directly place point mutations in the DNA of cells without DSBs (Komor et al., 2016; Gaudelli et al., 2017). Base editors are comprised of a Cas enzyme and a single-stranded DNA modifying enzyme for targeted nucleotide alteration. Approximately 25% of human pathogenic single nucleotide polymorphisms (SNPs) can be corrected using BE. The PE system has further diversified CRISPR gene editing capabilities to include all of the twelve types of transition and transversion mutations, including small insertions and deletions. Similar to BE, PE does not rely on establishing a DSB and instead utilizes an engineered reverse transcriptase that is fused to Cas9 nickase and a prime-editing guide RNA (pegRNA). The pegRNA contains both complementary sequences to the target site, which directs Cas9 to its target sequence, and a sequence that spells the desired sequence changes. By and large, PE has the potential to correct up to 89% of known genetic variants associated with human disease and to edit large genes which are not addressable using viral vectors with limited packaging capacity. Though BE and PE hold great potential for gene therapies, further characterization of BE and PE is needed to assess their off-target effects. Additionally, further evaluation of both methodologies in *in vivo* models is required (Komor et al., 2016; Gaudelli et al., 2017).

While the mechanism of the CRISPR/Cas9 system becomes increasingly better understood, the further development of safe and effective ways to package gene editing reagents as well as improved intracellular delivery methods are required to enable broader clinical applications (Yip, 2020; Zhang et al., 2021b). Delivery of biomolecular cargoes that encode for the transient expression of the Cas9 protein carry with them a number of barriers to clinical use, which current investigations seek to address. Specifically, the precise insertion or deletion of DNA can be directly related to the successful delivery of cargo to cells and the DSB repair mechanism utilized. With the % HDR, target DNA site selection, sgRNA design, Cas9 activity, and subsequent off-target effects significantly impacting the success rate of gene editing, optimization of these parameters remains the key to clinical viability of CRISPR/Cas9-mediated genome engineering (Liang et al., 2017).

When approaching the existing challenges to clinical translation outlined above, there are two key, interconnected components to consider: the gene editing cargo to be delivered and the mechanism of delivery. Well-established types of gene editing cargoes include Cas9-encoding DNA plasmids or messenger RNA (mRNA) constructs and Cas9 ribonucleoprotein (RNP) complexes. Each of these presents its own advantages and challenges. A Cas9 RNP complex consists of the Cas9 protein and a sgRNA. mRNA-based cargoes encoding Cas9 only require delivery to the cytosol for translation, whereas plasmids tend to be larger, more difficult to encapsulate, and must be trafficked to the nucleus for transcription. In contrast, while plasmids are relatively stable, mRNA presents with stability issues in physiological conditions. Protein-based cargoes such as Cas9 RNPs do not require transcription or translation, but the complex distribution of surface charges can make integration with certain delivery systems challenging. The ability to deliver multiple gRNAs via expression plasmid templates enables multiplexed gene editing. Limitations to this approach include a lower average editing efficiency when the guides are delivered as separate gRNA transcripts (8.2%) as opposed to a single gRNA array linking several gRNA transcripts (25%) as well as increased cell death (Kurata et al., 2018). Additionally, transfection of these plasmid-based CRISPR/Cas9 cargoes requires complex guide preparation that can result in increased off target effects due to their persistent expression compared to the shorter, more transient activity of pre-complexed RNPs (Liang et al., 2015).

Cargo encapsulation and cellular uptake mechanisms must both be considered in the design and selection of delivery systems. There are several approaches to temporarily porate the cell membrane, each with their own advantages and challenges. Methods to physically generate pores in the cell membrane *via* mechanoporation techniques, including microinjection (Crispo et al., 2015; Hruscha and Schmid, 2015; Martin-Martin et al., 2018), microfluidics/cell squeezing (Saung et al., 2016; Bridgen et al., 2017), and sonoporation (Helfield et al., 2016) are all currently under development. The most widespread delivery method is electroporation, whereby cells are exposed to an electrical field in order to create pores in the membrane that facilitate reproducible and efficient intracellular entry of biomolecules into cells (Deng et al., 2018; Kang et al., 2020). However, this method tends to stress cells considerably and is often associated with low post-transfection viability, potentially compromising its utility for some autologous cell therapies where limited numbers of donor cells can be harvested. Cells may also be porated by exploiting the thermoplasmonic properties of metal nanoparticles, which can cause localized heating and temporarily damage the cell membrane (Xiong et al., 2014). Other approaches to intracellular delivery do not require transient membrane poration. Specifically, non-plasmonic nanoparticles may be used to encapsulate and deliver intact CRISPR/Cas9 cargoes intracellularly. Supramolecular and lipid nanoparticle formulations are of particular interest as they can be engineered to bear positive surface charge and protect their cargo from degradation (Ping et al., 2011; Ashok et al., 2021). In addition, nanoparticles are scalable to synthesize and tunable in size, and are thus promising delivery vehicles for gene editing cargoes.

These non-viral intracellular delivery approaches are not without their challenges. Currently, viral vectors, which harness a virus’ natural ability to enter cells and to modify DNA, remain the delivery vehicle of choice for most clinical gene therapies. While existing viral vectors are effective in laboratory settings, they do not translate easily for many clinical applications due to limitations in their cargo carrying capacity (Wu et al., 2010) as well potential issues with immunogenicity and insertional mutagenesis due to the semi-random gene insertion mediated by these viral carriers (Nault et al., 2015). Non-viral vectors, including several nanoparticle-based systems, have more recently been identified as viable alternatives to viral vectors. Indeed, non-viral vectors have been shown to deliver Cas9/sgRNA plasmids *in vitro* with one study reporting a 47% successful transfection of plasmid in A374 cells, resulting in >67% suppression of tumor growth *in vivo* (Zhang et al., 2017a). Recent design considerations for non-viral delivery vectors have emphasized increasing gene delivery and expression efficiencies (Li et al., 2018a).

CRISPR/Cas9 has transformed the ease and precision of gene modification. While the rapid progress made in the efficiency and accuracy of CRISPR/Cas9 technology has provided new capabilities for establishing robust and durable therapeutic interventions, significant barriers to broader clinical adoption persist. This review aims to summarize current and emerging bioengineering approaches used to direct the transport of CRISPR/Cas9 gene editing tools into targeted cells. We focus on two engineerable components: the delivery vehicle and the gene editing cargo it carries. An understanding of these tools will help to provide an overview of the contemporary CRISPR/Cas9 clinical landscape, the challenges that lie ahead on the road to therapeutic gene editing using CRISPR/Cas9, and the considerations required when selecting both CRISPR/Cas9 cargo and the delivery vehicle to be used.

## Gene editing cargo

Successful clinical application of CRISPR/Cas9-based therapeutics requires both accurate binding to the targeted sequence in the host genome (Liang et al., 2017) and efficiency in the repair mechanism following the formation of Cas9 endonuclease-induced DSBs. There are an increasing number of CRISPR-based cargo options currently being optimized to address these challenges. The DSBs induced by the Cas9 protein are an essential feature of the CRISPR/Cas9 system as they enable the correction of defective endogenous genes. The repair mechanism subsequently applied to DSB sites primarily determines the mode of gene editing *via* either gene knockout, deletion, correction, or insertion. Repair of DSBs follows one of two mechanisms: NHEJ using protein factor re-ligation, or HDR by a homologous DNA repair template. NHEJ-mediated repair is less versatile and more prone to unwanted off-target deletions, whereas HDR precisely repairs the DSB but is cell cycle dependent (limited to the late S- or G2-phase). As a result, many attempts to create clinically applicable CRISPR/Cas9 cargo are focused on further increasing the efficiency and incidence of HDR (Chu et al., 2015; Maruyama et al., 2015; Liang et al., 2017; Li et al., 2018a). A deeper understanding of the factors that determine the ratio of HDR to NHEJ remain largely unknown, however, a study by Kato-Inui et al. (2018) has revealed that modified sgRNAs and Cas9 variants may be used to enhance HDR, suggesting that modifications to the traditional CRISPR/Cas9 system could optimize the HDR:NHEJ ratio. The modification of Cas9 has further provided an opportunity to overcome several significant HDR-related limitations of CRISPR/Cas9 in the site-specific correction of human hematopoietic stem cells, which exhibit lower HDR:NHEJ. For example, Kohn and colleagues found that a modified Cas9 with reduced nuclease activity transiently increased the number of cells in the HDR favored S/G2 phase, resulting in a four-fold increase in the HDR:NHEJ ratio. These insights ultimately inform the rational design of CRISPR/Cas9 gene therapies where HDR:NHEJ is critical (Lomova et al., 2018).

### Single guide RNA

Recent research has been largely focused on optimizing guide RNAs that are delivered in conjunction with the Cas9 nuclease. Traditional guide RNA constructs can be divided into two separate RNA strands: Target-specific CRISPR RNA (crRNA) and target-nonspecific trans-activating CRISPR RNA (tracrRNA), which hybridize to bind the targeted DNA sequence for mutagenesis (Latorre et al., 2016). Jinek et al. (2012) first combined these transcripts into a programmable single guide RNA after elucidating the relationships between tracrRNA, crRNA and Cas9 through a series of electrophoretic mobility shift assays, which illustrated that tracrRNA must recognize the targeted DNA strand once correctly positioned by the crRNA. Furthermore, introduction of specific chemical modifications to this nucleic acid-based guide molecule has been shown to affect Cas9 activity (Jinek et al., 2012).

**FIGURE 1:**
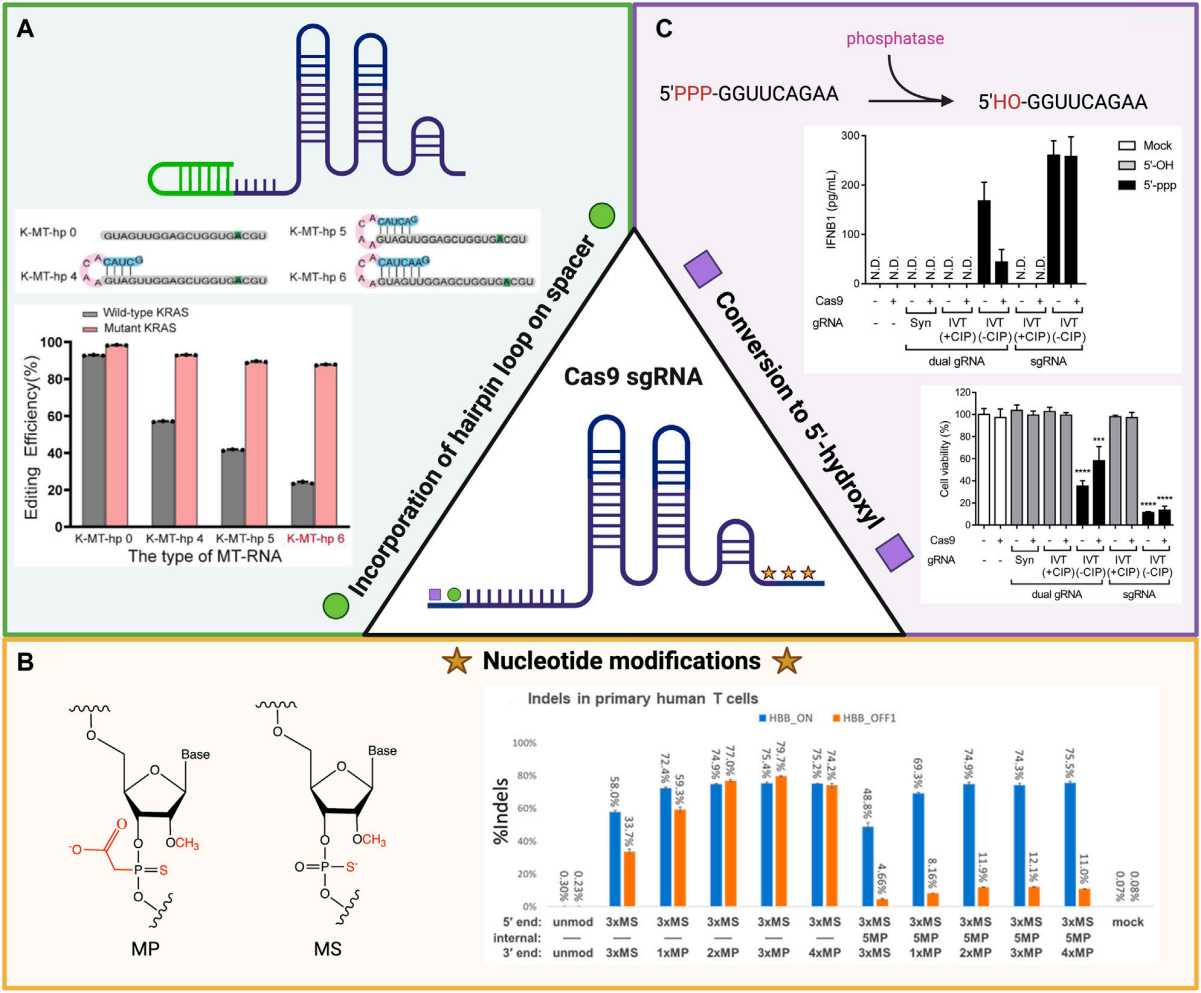
Schema of the chemical modifications that affect Cas9 editing efficiency and specificity of single guide RNA (sgRNA) to target sequences. **(A)** The incorporation of hairpin loops in sgRNA increases the selective *in vitro* editing efficiency of the mutant KRAS gene over the wildtype version in comparison to linear sgRNA in MCF-7 cells. Image reused with permission from ref (Liang et al., 2022). Copyright 2022 American Chemical Society. **(B)** Specific chemical modifications (left) to nucleotides on both the 5′ and 3′ ends of synthesized sgRNA have increased on-target and off-target gene disruption in primary human T cells for the HBB gene (right). Image reproduced from ref (Ryan et al., 2022) with permission from American Chemical Society. License CC BY-NC-ND 4.0 https://creativecommons.org/licenses/by-nc-nd/4.0/. **(C)** Conversion of the 5′-triphosphate group to a 5′-hydroxyl group on the 5′ end of sgRNA through the addition of calf intestinal phosphatase has led to a decrease in IFNB1 immune response and increase in cell viability when transfected with Cas9 ribonucleoprotein (RNP). Image reproduced from ref (Kim et al., 2018) with permission from Genome Research License CC BY-NC 4.0 https://creativecommons.org/licenses/by-nc/4.0/. Schematics created using BioRender.

Hairpin loops are common secondary structures within RNA molecules and can regulate gene expression in either a *cis* or *trans* manner. A *cis*-acting hairpin influences expression within the RNA molecule, while a trans-acting hairpin affects other RNA molecules and pathways (Svoboda and Di Cara, 2006). When comparing the editing efficiencies of linear versus hairpin-engineered sgRNAs of different lengths in MCF-7 cells, Liang et al. (2022) demonstrated that the hairpin structures had a higher selectivity for editing the mutant sequence of the KRAS gene target over the wildtype sequence (Figure 1A). Kocak et al. (2019) purposefully incorporated hairpins within the spacer sector of sgRNA (hp-sgRNA) and compared these modified constructs to non-structured sgRNA of the same size while monitoring the editing activity of the Cas9 protein at off-target sites in HEK 293T cells. They hypothesized that the hairpin structure would provide a steric barrier that only allowed editing for specific, on-target sites, and observed *via* sequencing analysis reduced off-target activity with hp-sgRNA. These data indicate that the addition of a secondary structure improved the specificity of the Cas9 RNP complex. The improved specificity of the hp-sgRNA for the Cas9 complex was confirmed when compared to sgRNAs with a truncated spacer sequence, and unmodified sgRNA). Despite the advantages of hp-sgRNAs, the possible cytotoxic effects have not yet been fully defined. The mechanism of hp-sgRNAs’ action on cell viability remains a critical barrier to their clinical use (Kocak et al., 2019; Moon et al., 2019; Hu et al., 2021).

While molecular modifications to sgRNA aim to improve the specificity of gene editing and enhance the safety of these therapies, they do not address the problems with low efficacy conferred by the susceptibility of RNA to degradation. Strategies to increase the stability of RNA in the presence of degrading ribonucleases and enhance the degree of binding to complementary sequences have recently incorporated alterations to portions of the sgRNA sequence. Hendel et al. (2015) modified the final three nucleotides on both the 5′ and 3′ ends of three separate sgRNA molecules and compared their enzymatic activities *in vitro* for both primary human T cells and CD34^+^ hematopoietic stem and progenitor cells. The modifications tested included incorporating 2′-*O*-methyl (M), 2′-*O*-methyl 3′ phosphorothioate (MS) or 2′-*O*-methyl 3′thioPACE (MSP) into the nucleotides. These changes were not found to diminish endonuclease activity *via* T7 assay, but MS and MSP did increase the frequency of indels based on tracking of indels by decomposition (TIDE) analysis of polymerase chain reaction (PCR) amplicons from the different sgRNA target sites (Hendel et al., 2015). An increase in insertions and deletions without a decrease in level of activity demonstrates how these modifications improve the nuclease’s editing efficiency. Jing et al. (2021) included the same MS modification to their sgRNA and observed a 15% higher knock-in efficiency compared to unmodified sgRNA when attempting to knock-in predetermined template plasmids into primary human T cells. Ryan et al. (2022) similarly engineered the MS and MP nucleotide modifications to their synthesized sgRNA structures and observed increased indel editing for the HBB gene within human primary T cells. By incorporating these alternations within the internal structure of these molecules, they were able to decrease the percentage of off-target indels thus increasing the sgRNA selectively (Figure 1B) (Ryan et al., 2022).

While these data are promising, such chemically modified nucleic acid sequences may induce unwanted and unpredictable immunological responses. Kim et al. (2018) reported that incorporation of a 5′-triphosphate group on sgRNA could promote innate type 1 interferon-mediated immune responses. The phosphatase-mediated removal of this nucleoside triphosphate group led to an increase in cell viability and reduction in the previously associated immune response (Figure 1C) (Kim et al., 2018).

### Plasmid DNA

Plasmid DNA (pDNA) is a well-established cargo for transfection and gene editing studies. Based on the method of adaptive immunity within prokaryotes, circular or linear DNA is delivered to a target cell and then trafficked into the nucleus for the expression of a gene of interest (Bower and Prather, 2009). Plasmids are advantageously stable within the cellular microenvironment and their potential for genomic integration when designed with cassettes encoding for transposon systems such as Sleeping Beauty or PiggyBAC may lead to longer lasting genomic changes (Cicek et al., 2019). However, pDNA has been shown to induce lower editing efficiency than RNPs when delivered *via* electroporation (Kim et al., 2014). Engineered modifications to pDNA constructs are under investigation to increase the overall rate of transfection and minimize cytotoxic effects. Zamolo et al. (2020) monitored green fluorescent protein (GFP) expression for a library of peptide dendrimers and discovered one (Z22) that combined a hydrophobic core with a highly branched configuration that helped transfect an ‘all-in-one’ CRISPR/Cas9 pDNA vector into 62.5% of HEK 293 cells and 47.3% of HeLa cells while also showing no reduction in cell viability (Zamolo et al., 2020). Similar supportive structures may enhance transfection in primary cell types.

The relatively larger size (∼9.3 kb) and higher molecular weight of pDNA constructs represent major barriers to efficient delivery and intracellular trafficking (Wang et al., 2018a). Since transcription of pDNA requires localization to the nucleus, these cargoes are susceptible to off-target editing by the transcribed Cas9 endonuclease (Miller and Siegwart, 2018). These extraneous edits are often unpredictable and can be disastrous to targeted cells. Tissue- and cell-specific promoters and enhancers that overcome these issues are being investigated to enable clinical-scale gene editing (Cicek et al., 2019).

One interesting analog to pDNA expression cargoes are minicircle DNA vectors (mcDNA). Similar to pDNA, this episomal DNA architecture is replicated in bacteria from a parental plasmid, but with the bacterial backbone and antibiotic resistance sequences removed to reduce the size of the vector (Kelly et al., 2021). The smaller size of mcDNA constructs makes them attractive for applications involving the delivery of larger gene expression cassettes. For example, Eusébio and colleagues observed that mcDNA encoding tumor suppressor gene p53 yielded more efficient transfection and sustained expression compared to pDNA vectors in HeLa cells (Eusébio et al., 2021). Kelly et al. (2021) utilized mcDNA both for the combined Cas9 and sgRNA-expressing vector as well as the donor templates designed for HDR or homology-independent targeted integration (HITI)-mediated insertion of a tdTomato reporter into the AAVS1 safe harbor locus of three human cell lines HEK 293T, HeLa, and PC3. Successful knock-in integration was measured through junctional PCR analysis and fluorescence microscopy. An added benefit to incorporating Cas9-encoding cargoes into smaller form factors is their higher resistance to degradation by hydrodynamic shearing forces relative to linear DNA when delivered via non-viral vectors. These potentially disruptive forces have the ability to affect the cargo as it is delivered to the target cells, and were modeled through nebulization processes to reflect clinical relevance (Catanese et al., 2012) While the effect that the removal of bacterial backbone sequences has on immunogenicity remains to be fully determined, there is evidence of a decrease in pulmonary inflammation when mcDNA is delivered to the lung in comparison to pDNA (Munye et al., 2016; Florian et al., 2021). To parse out the optimal mcDNA configuration for the CRISPR/Cas9 system, more studies are required that directly compare transfection efficiency and cytotoxicity across several types of nucleic acid-based cargoes.

### Messenger RNA

To diversify possible payloads that do not require genomic integration, mRNA is often leveraged. Cas9-encoding mRNA transcripts are shorter (∼4.5 kb) than DNA plasmids and they are already spliced and ready for cytosolic translation (Kenjo et al., 2021). These cargos tend to display lower off-target activity and are well suited for clinical applications as indicated by their use in the recent COVID-19 vaccines. For example, Liang et al. (2015) observed a 2-fold lower rate of off-target cleavage when Cas9 was delivered to HEK 293FT cells as mRNA compared to cells transfected using pDNA (Liang et al., 2015).

Various alterations to the mRNA construct are under investigation, including modifications that result in increased cell viability and transfection efficiency. One well-known and naturally occurring structural modification is to replace uridine with pseudouridine (Kariko et al., 2008). Modified mRNA was translated to a greater extent at each measured time point than unmodified mRNA in rabbit reticulocyte lysates, these results could not be replicated for wheat germ extracts and *E. coli* lysates (Kariko et al., 2008). This outcome postulates that the benefits of cargo manipulation are most likely cell type dependent. Vaidyanathan et al. (2018) built on these results and tested a combination of four additional modifications on a mRNA transcript for Cas9 specifically. A uridine-depleted transcript with 5-methoxyuridine (without HPLC purification) was selected as the preferred candidate for further *in vitro* and *in vivo* testing as it balanced a reduced innate immunogenic response with the feasibility for mass production (Vaidyanathan et al., 2018). Although these initial results are promising, continued optimization of mRNA constructs will likely be required to further reduce immunogenicity and increase molecular stability before widespread clinical translation is achievable.

Importantly, when mRNA transcripts are used in CRISPR/Cas9 applications, the endonuclease is expressed transiently. This is advantageous even when multiple doses may be required to maintain a significant intracellular level of Cas9 in non-renewing cell populations, because prolonged presence of the protein is associated with cytotoxicity, off-target cutting, and immune responses (Knopp et al., 2018; Kowalski et al., 2019). Transient expression of Cas9 mitigates these undesired effects. The use of mRNA transcripts in *ex vivo* applications may therefore minimize patient immunogenicity by ensuring that corrected cells are cleared of Cas9 before they are administered to patients, and reduce the time immunosuppressants are required for *in vivo* treatments (Crudele and Chamberlain, 2018). While the transient expression of mRNA has thus far been considered favorable in developing CRISPR/Cas9 gene therapies, the need for further control over protein expression has pushed the creation of programmable, small-molecule-responsive RNA binding proteins to control expression of proteins from RNA-encoded genetic circuits (Wagner et al., 2018). External regulation of protein expression in BHK-21 cells and a mouse myoblast cell line, C2C12, was achieved by leveraging a small-molecule-regulated safety switch that cleaves the RNA circuit when a small molecule is introduced. While to the authors’ knowledge this technology has not yet been directly applied to the CRISPR/Cas9 system, controllable, self-replicating mRNA may provide additional control over transiently expressed mRNA (Wagner et al., 2018; Pandelakis et al., 2020). Other characteristics of mRNA constructs for CRISPR/Cas9 are its high molecular weight (>330 kDa), high degree of hydrophilicity and anionicity, and low stability (Miller and Siegwart, 2018). These factors collectively impair transport of mRNA molecules to across membranes and stability in different cellular microenvironments, thus contributing to the lower transfection efficiencies observed when delivery is attempted without the appropriate packaging.

### Ribonucleoprotein complexes

Cas9 RNPs composed of the recombinant Cas9 protein complexed with a sgRNA, often referred to as protein-based Cas9, have been applied to accomplish both efficient and on-target genome editing. Like mRNA, protein-based cargoes like RNPs ensure transient endonuclease activity and reduced likelihood of off-target effects. However, the sgRNA-Cas9 complex exhibits a better stability profile compared to mRNA (Tang et al., 2021). Advantages of RNPs include lower off-target editing, fast action, and transience of the protein. However, the higher reagent costs associated with these protein-based cargoes often precludes large scale studies, and the size of the Cas9 protein (160 kDa) often limits delivery efficiency. However, recent advances in encapsulating Cas9 into polymer nanoparticles have begun to offer solutions for delivering Cas9 RNP for *in vivo* genome editing (Chen et al., 2019).

Efforts to further increase the efficiency and precision of RNPs for clinical translational studies primarily target increasing the incidence of HDR. A recent study revealed two successful approaches for improvement across multiple genomic loci in diverse cell types (Nguyen et al., 2020). The first involved addition of truncated Cas9 target sequences to the ends of the HDR template, which then interact with RNPs to shuttle the template to the nucleus. This approach has been shown to enhance HDR efficiency fourfold. The second involved using polyglutamic acid nanoparticles to stabilize Cas9 RNPs to achieve a two-fold increase in editing efficiency. In addition to increased efficiency, these modifications also resulted in increased stability, reduced toxicity, and enabled lyophilized storage.

The inclusion of modified donor DNA has also shown promise in further promoting HDR. The co-delivery of Cas9 RNPs with donor DNA exhibiting modifications close to the cleavage site showed improved integration efficiency in HEK 293T cells. Specifically, phosphorothioate modifications have been applied to protect the ends of the donor DNA and improve editing efficiency (Liang et al., 2017). In addition, incorporating targeting vectors with 3′ overhangs at both ends of the donor DNA was shown to increase HDR efficiency significantly in mouse embryonic stem cells using specific modulating targeting vectors (Hirotsune et al., 2020). Both methods increase the occurrence of HDR when delivering protein based Cas9 and efficiently improve editing precision.

The CRISPR/Cas9 system has the ability to transform approaches to treating genetic diseases. The appropriate cargo must be selected for any particular goal or application. Each form of Cas9 endonuclease, be it a pDNA construct, mRNA construct, or protein, comes with its own benefits and limitations. Recent studies have balanced the optimization of these subtypes of cargo through various chemical manipulations to improve transfection and editing efficiencies and reduce immunological responses and off-target effects that hinder possible clinical applications. Improving mechanisms of delivery must go hand-in-hand with the optimization of CRISPR/Cas9 cargoes if the goal of clinical translation is to be achieved.

## Physical and energetic methods of delivery

While the optimization of CRISPR/Cas9 cargoes is essential for successful gene editing, delivery of these optimized cargoes to target cells and tissues represents an enormous barrier to effective clinical gene therapies. In recent years, the toolkit of available methods for intracellular delivery has largely expanded, and the advantages and limitations of each have been better elucidated and addressed. The methods used to deliver the gene editing cargo can be classified into physical, energetic, and particle-based delivery. Common techniques for delivery include mechanoporation and micron/nanoscale structure-mediated membrane penetration, electroporation, and acoustoporation.

### Mechanoporation and micron/nanoscale structure-mediated membrane penetration

**FIGURE 2:**
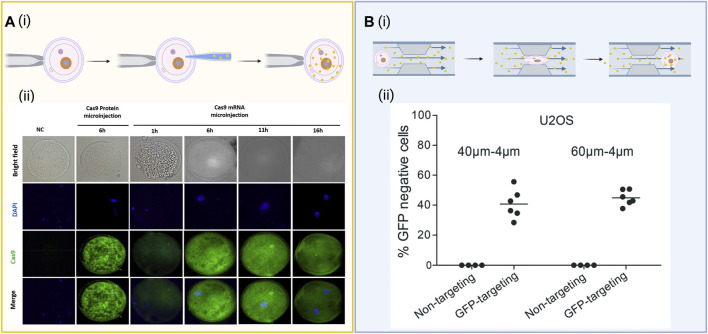
Diagrams of common biophysical delivery methods. **(A)** (i) Schematic of a microneedle directly injecting gene editing cargo into the nucleus of a cell. (ii) Evaluation of Cas9 expression over 16 h when microinjected as messenger RNA (mRNA), the protein itself (positive control), and a nuclease-free injection of water (negative control) under both brightfield and immunofluorescent staining. Images reproduced from ref (Li et al., 2021) with permission from Theriogenology. License CC BY-NC-ND 4.0 https://creativecommons.org/licenses/by-nc-nd/4.0/. **(B)** (i) Cell squeezing involves passing the cells through a micrometer-sized constriction at high speeds to form transient pores along the cellular membranes. The cargo can diffuse into the cell through the pores before the membrane reassembles. (ii) Flow cytometry data for expression of green fluorescent protein (GFP) after delivery of a Cas9 ribonucleoprotein (RNP) complexed to either a GFP-targeting or nontargeting guide RNA (gRNA) after GFP-expressing U20S cells were flowed through a microfluidic device with either 40–4 µm or 60–6 µm channels designed for cell squeezing. Images reproduced from ref (Uvizl et al., 2021) with permission from the Royal Society of Chemistry. License CC BY 3.0 https://creativecommons.org/licenses/by/3.0/. Schematics created using BioRender.

The physical disruption of the cell membrane is a very common method of permeabilizing cells to cargoes, which do not readily enter cells on their own. Membrane disruption can be achieved using a variety of micron and/or nanoscale structure-mediated approaches. Microinjection, as illustrated in Figure 2A a type of mechanical transfection that uses a micrometer-sized capillary to inject gene editing cargo into cells. It is favored for its practicality in single-cell applications and its precision in the mechanical delivery and retrieval of biomolecules to and from the cell nucleus to enable rapid gene editing (Wu et al., 2015). Using a microscope and a microneedle (0.5–5.0 μm diameter), plasmid DNA, mRNA, or Cas9 protein can be directly injected into the membrane of a cell of interest *via* microinjection. This technique has been used in recent years to generate genetically-modified animals including sand flies, sheep zygotes, and zebrafish (Crispo et al., 2015; Hruscha and Schmid, 2015; Martin-Martin et al., 2018) with specific-site mutations into embryos, but has often resulted in the generation of mutant embryos with several cells carrying mutations (Le et al., 2021). DNA and mRNA are the most commonly used cargoes for microinjection delivery, where DNA is able to freely transcribe and translate its components in the nucleus and mRNA is injected directly into the cytoplasm to be translated by the cell. For example, Chuang et al. (2017) utilized microinjection of DNA that encodes both Cas9 and sgRNA directly into the nucleus to eliminate the transcription reactions that occur *in vitro*. Raveux et al. (2017) used microinjection of CRISPR mRNA components into the cytoplasm to enable the sgRNA to bind to Cas9 and enter the nucleus of the cell. Li et al. (2021) investigated the effects of the timing of microinjection on embryo development and the gene targeting efficiency of the CRISPR/Cas9 system to disrupt the interleukin 2 receptor subunit gamma (IL2RG) locus using porcine *in vitro* fertilization (IVF) and somatic cellular nuclear transfer (SCNT) derived embryos. To evaluate the Cas9 mRNA translation time in the porcine embryos post-microinjection, Cas9 protein expression was detected at low levels after 1 h and expressed higher levels after 6 h, as illustrated in Figure 2A. Though highly effective at delivery to a cell of interest, microinjection requires a skilled technician to maintain cell viability, produces low throughput of the cargo, is labor intensive and time consuming, and is generally limited to small scale *in vitro* studies.

Mechanoporation may also be achieved by directing cells to nanoscale cell-penetrating structures. Specifically, nanoneedle arrays have been used to transfect larger numbers of cells *via* direct penetration of the cell membrane to deliver biomacromolecules adsorbed to the nanoneedle surface. This method, leveraging RNP-adsorbed arrays of silicon nanoneedles 200 nm in diameter, was found to induce gene editing efficiencies of up to 32%, and allowed for the transfection of adherent cells while in monolayer rather than in suspension (Yamagishi et al., 2019). Melosh and colleagues recently demonstrated a magnetic nano-electro-injection (MagNEI) platform that is used to transfect primary human T cells efficiently (Tay and Melosh, 2021). This method involves the localization of electric fields generated from hollow nanochannels to open pores transiently on the membrane of cells, allowing DNA to enter. Once DNA is inside the cell, magnetic forces are applied *via* Dynabeads™ (ThermoFisher) to enhance nuclear transport, thus resulting in enhanced DNA transfection. These magnetic forces also accelerate the membrane repair and help to sustain cell proliferation and gene expression throughout the transfection process through the promotion of actin cytoskeletal remodeling (Tay and Melosh, 2021). Another method, deterministic mechanoporation, achieves single-cell delivery through the utilization of aspiratory flows and a sub-micrometer-scale needle within individual wells on a large array of captures sites. The concave wells are fabricated to be cell type size specific and the aspiratory flows are regulated to ensure that the tension on the plasma membranes facilitates needle penetration, yet does not deform the target cell. After a transient single poration site is created in the membrane, small to large cargo can be delivered to the target cells *en masse*. This approach has successfully transfected Jurkat (88%), K562 (49%), and primary human T cells (82%) with GFP plasmid while maintaining high cell viability. Although a high throughput approach, the requirement to treat cells *ex vivo* is not suitable for all cell types (Dixit et al., 2020). These nanostructure-mediated membrane penetration methods streamline injection-based CRISPR/Cas9 gene editing for adherent cells and increase throughput compared to microinjection for suspension-type cells tethered to a culture dish. These delivery strategies are still being investigated for clinical translation.

Cell squeezing, another microfluidic-based biophysical mechanoporation technique, passes cells at high speeds through micrometer-sized constrictions, disrupting the plasma membrane and enabling the delivery of various cargoes through the cytosol of numerous cell types (Sharei et al., 2013), illustrated in Figure 2B. Saung et al. (2016) found that a 4 μm wide constriction is effective for delivery of cargo to primary human T-cells that have an average diameter of 6.7 μm, whereas a 6–7 μm wide constriction is better optimized for cell lines between 10.8 and 12.3 μm such as BxPc3 and PANC-1. In addition, Han et al. (2015) effectively delivered CRISPR into difficult-to-transfect SU-DHL-1 lymphoma cells *via* cell squeezing using a microfluidic device made of diamond-shaped polydimethylsiloxane (PDMS) pillars with a constriction width of 4 µm. According to the study, a sharp angle of deformation preserved cell viability better than a curved constriction. Using the same device, they managed to deliver an enhanced green fluorescent protein (EGFP) reporter plasmid to ∼30 and 50% of SU-DHL-1 lymphoma cells and AB 2.2 mouse embryonic stem cells, as well as knockout EGFP in MDA-MB-231 (human breast cancer) and SU-DHL-1 cell lines. Another study by Han et al. (2017) demonstrated delivery of a RNP cargo configured to knock out EGFP using a microfluidic device to mechanically transfect EGFP-expressing SK-BR-3 cells, MDA-MB-231 cells, SU-DHL-1 cells, and human primary T cells. The study found maximum knockout efficiency occurred at 2 µM RNP and demonstrated a mutation frequency for the MDA-MB-231 cells, SU-DHL-1 cells, and human primary T cells to be 43, 47, and 33%, respectively. The device achieved delivery efficiencies of roughly 40% for both RNPs and Cas9 plasmid constructs, with plasmids generating an off-target mutation rate of 4.7% compared to 0.8% for RNPs. Similarly, cell-squeezing devices with progressively narrow channels either 40–4 µm or 60–6 µm in diameter were shown to effectively deliver functional RNPs targeting the GFP gene in a stably-expressing GFP reporter U20S cell line, inducing ∼40% knockout of the GFP gene (Uvizl et al., 2021) (Figure 2B). Similarly, Bridgen et al. (2017) demonstated the ability to use cell squeezing in the delivery of Cas9 RNPs to primary human T cells and CD34^+^ hematopoietic stem and progenitor cells to edit the CCR5 and B2M loci with little detectable impact on cell differentiation, proliferation, and function, further establishing cell squeezing as a viable method for cell therapy manufacturing.

Though cell squeezing *via* microfluidic devices demonstrates transfection efficiency to various cell types, it presents additional challenges. While delivery *via* cell squeezing is well documented, the repair mechanism of the plasma membrane must be understood as it may have detrimental effects on cell viability. Sharei et al. (2014) studied membrane recovery kinetics by examining rate of repair while varying buffer composition. They demonstrated that recovery is an active, calcium-mediated process as it cut recovery time of HeLa cells from over 3 min in phosphate buffered saline (PBS) alone to 15–30 s with the addition of calcium. By understanding the relationships behind the factors that affect membrane repair mechanisms, poration methods may be improved to retain high transfection while increasing cell viability. Various studies also encounter device clogging, inverse proportionality between cell viability and transfection efficiency over time, as well as the inability to deliver nucleic acids (Chakrabarty et al., 2021). Additionally, this method of delivery is optimized for *in vitro* work and cannot be easily adapted for *in vivo* applications.

Another technique of microfluidic-based mechanoporation called hydrodynamic manipulation can deliver large macromolecules *in vivo* by injecting a liquid solution intravenously at extremely high volume and pressure. This sudden increase in volume induces the temporary generation of pores in the vasculature, allowing the large macromolecular payload to reach the target tissue. This technique is commonly paired with other delivery techniques as it excels in distribution of deliverables to *in vivo* tissues but does not necessarily have a method to bypass cellular membranes themselves. Hydrodynamic injections *via* the tail vein in mice have successfully delivered plasmids carrying Cas9 and gRNA into the heart, lungs, liver, and kidney tissue. Presently, this method is restricted to use with small animal models due to the large starting injection volume necessary (∼10% body weight of the mouse). As such, it is currently not appropriate for human applications, although research is ongoing to optimize this technique for larger animal use (Chakrabarty et al., 2021). Deng et al. (2018) investigated delivery of various cargos such as protein, siRNA, CRISPR/Cas9, plasmid DNA, and DNA nanomaterials to different cell types using a hydrodynamic delivery platform termed inertial microfluidic cell hydroporator (iMCH) (Deng et al., 2018; Kang et al., 2020). The iMCH focuses cells into a channel center, leading them into a T-junction where the membranes are rapidly deformed and rendered transiently porous to allow the uptake of the nanomaterials into the cytoplasm. This method of hydroporation was found to maintain high cell viability and achieved a COL11A1 gene knockdown efficiency of over 80% in A2780cis cells with the successful delivery of the CRISPR/Cas9 system (Deng et al., 2018) and delivers nanomaterials to the cell while overcoming some of the toxicity challenges of earlier nanocarrier or membrane disruption techniques (Deng et al., 2018; Kang et al., 2020). This system was further developed to establish mixing in conjunction with the transient deformation of the cell membrane by incorporating spiral vortex flows at the T-junction of the microfluidic device to facilitate both passive diffusion and convection-based rapid solution exchange across the membrane of processed cells (Hur et al., 2020). The successful delivery of gold and silica nanoparticles, dextran, and mRNA to MDA-MB-231 human epithelial breast cells at efficiencies of up to 96.5% with cell viability of up to 94.5% was demonstrated (Kang et al., 2020). Most recent updates to this system leverage droplet microfluidics in the channel, reducing cargo consumption, scaling throughput and reducing clogging to near-zero when treating primary human T cells (Joo et al., 2021). The continued scaling and development of this system has established hydroporation as a simple, efficient, high throughput, low-cost, and clinically applicable intracellular delivery system (Deng et al., 2018; Kang et al., 2020).

### Electroporation

**FIGURE 3:**
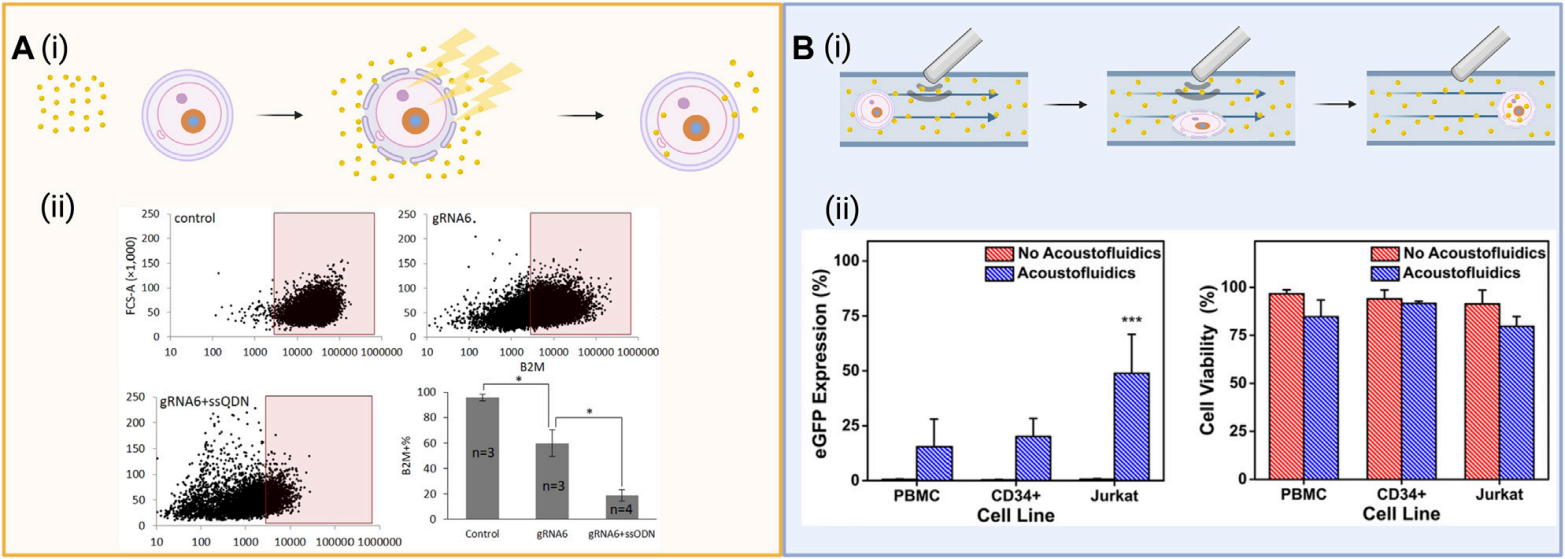
**(A)** (i) Schematic of electroporation which entails exposing cells in solution with the cargo to a strong electric field exceeding the membrane’s capacitance. This opens pores in the cell membrane through which the cargo can diffuse into the cell. (ii) Intracellular delivery of functional Cas9-sgRNA RNPs. Cas9-sgRNA ribonucleoprotein (RNP) complexes targeting the B2M locus were delivered into human mesenchymal stem cells using tube electroporation, reducing surface expression of B2M from 95.6 to 59.9%. Figures reused from ref (Xu et al., 2018) with permission from Scientific Reports. License CC BY 4.0 https://creativecommons.org/licenses/by/4.0/ **(B)** (i) Schematic of acoustoporation, showing ultrasonic acoustic waves oscillate microbubbles that burst under high pressures, perforating the membrane. The transient pores formed allow cargo to diffuse into the cell. (ii) eGFP expression and cell viability 72 h after acoustofluidic delivery of an eGFP-expressing plasmid to Jurkat, peripheral blood mononuclear cells (PBMC), and CD34^+^ hematopoietic stem and progenitor cells (HSPCs). Figure reused from ref (Belling et al., 2020) with permission from Proceedings of the National Academy of Sciences of the United States of America. Schematics created using BioRender.

Electroporation applies a strong electric field across a cell membrane, which exceeds the membrane’s capacitance, to transiently open nanometer-sized pores as illustrated in Figure 3A. The increase in permeability allows large biomolecules that would otherwise be rejected to enter the cell (Gehl, 2003). Electroporation can readily deliver difficult-to-manipulate cargoes to a wide number of cell types, and is often most effective for immune cells and stem cells. Due to its simplicity and efficacy, electroporation is currently one of the most commercially available and attractive non-viral delivery methods for gene editing cargo (Qin and Wang, 2019).

Electroporation has been shown to be successful in *vitro* and *ex vivo* applications, for the delivery of RNPs, DNA, and mRNA for both knock-in and knock-out of target sequences (Yang et al., 2018). Alghadban et al. (2020) reported improvements in mutagenesis efficiency for the generation of ssODN repair templates and a higher rate of embryo survival and development when delivering CRISPR/Cas9 systems as RNPs *via* zygote electroporation. Another group achieved viable embryos and high CRISPR/Cas9 entry into the cells using RNAs that were electroporated into zygotes (Hashimoto and Takemoto, 2015) *via* nucleofection, a specialized form of electroporation that does not necessitate breaking down the nuclear envelope, to mouse spermatogonial stem cells to correct a cataract-inducing mutation (Wu et al., 2015). Cas9 RNPs are the preferred cargo for delivery *via* electroporation because they are more stable than their pDNA and mRNA counterparts. Though the processes of applying an electric field to cells can effectively deliver cargo, some electroporated cells may be damaged due to excessive heat exposure, ionic imbalances, and changes in pH. Notably, large plasmids can cause a decrease in viability in comparison to smaller plasmids by increasing permeabilization levels and duration in cells resulting in the prevention of the cells from resealing; standard electroporation buffers can cause substantial cell death, and uneven electric field distribution can cause liquid/air interfaces inside cuvettes where cells are housed that lower transfection efficiency (Lesueur et al., 2016; Cao et al., 2019). In an effort to further characterize the impact of electroporation on cell viability for the successful application to cell-based therapies, DiTommaso et al. (2018) comparatively analyzed microfluidic cell squeezing against electroporation by investigating disruptions in the expression profiles of key functional transcripts of human T cells. The study found that though both methods efficiently edited the cells, microfluidic cell squeezing had minimal transcriptional responses, showed undiminished effector responses, and therapeutic potential *in vivo* in comparison to electroporation (DiTommaso et al., 2018). This study highlights the importance of understanding the effects of intracellular delivery methods and that further optimization of electroporation techniques may benefit research and clinical applications.

To address some of the disadvantages of the electroporation method, Xu et al. (2018) developed a modified tube-shaped cuvette that is less prone to bubble formation. They applied this technique to knock-out β2-microglobulin (B2M), a component of major histocompatibility complex (MHC) class I molecules, in primary human messenchymal stem cells, reducing expression by 80.2% through the delivery of Cas9/gRNA RNP with an ssODN introducing a frameshift mutation through single base insertion (Xu et al., 2018), as well as reducing surface expression of the protein in mesenchymal stem cells from 95.6 to 59.9% (Figure 3A). Cao et al. (2018) developed a nanostraw electroporation platform to enhance the local electric field and decrease the operating voltage and bubble formation. This method of nanoelectroporation allows for spatial control of the cells in a smaller nanostructure interface that enhances the local electric field. The group reported 90% cell viability and 85% mRNA transfection efficacy (Cao et al., 2018). In an effort to overcome the challenges of electroporation outlined above, Ding et al. (2017) used a hybrid microfluidic electroporation device to create rapid mechanical deformation of the cell membrane by cell squeezing in combination with electric-field driven transport to efficiently increase DNA expression in HeLa cells within 1 h of treatment, demonstrating the power of combining and developing these delivery methods further. Additionally, Yang and colleagues leveraged nanopores present on commercially available polycoarbonate water filtration membranes to design an affordable nanopore electroporation (nanoEP) method. Optimization of these nanoEP devices resulted in the gene editing of the PPIB gene in about 25% HeLa and Jurkat cells using Cas9 RNPs and up to 95% viability after transfection (Cao et al., 2019).

Similarly, Roth et al. (2018) investigated non-viral genome targeting methods by co-electroporating human primary T cells with CRISPR/Cas9 RNP complexes and linear double stranded DNA (dsDNA) HDR templates designed to introduce an N-terminal GFP fusion in the housekeeping gene RAB11A to reduce the toxicity associated with the dsDNA template (Roth et al., 2018). According to the study, this method of electroporation resulted in up to 50% GFP expression in human CD4^+^ and CD8^+^ T cells, is highly efficient, maintains high cell viability, and provides preclinical evidence of therapeutic engineering of primary human immune cells. Stadtmauer et al. (2020) reported a Phase I clinical trial assessing the safety and feasibility of CRISPR/Cas9 gene editing in patients using electroporation. T cells were isolated from the blood of patients with cancer and CRISPR/Cas9 RNP complexes loaded with three sgRNAs were electroporated into the normal T cells, resulting in the successful gene editing of the TRAC, TRBC1, TRBC2, and PDCD1 loci (Stadtmauer et al., 2020). While preliminary results from the trial demonstrated the safe and feasible use of the CRISPR/Cas9 system, these studies soley utilize *in vitro* and *ex vivo* methodology, respectively. Recent efforts to apply electroporation for delivery of Cas9-mediated systems *in vivo* have yielded successful gene editing in skin stem cells in mouse models. Through the application of electroporation on mouse tail skin, Wu et al. (2017) restored C7 function in Recessive Dystrophic Epidermolysis Bullosa (RDEB) mice and observed an increase in epidermal-dermal adhesion from 30 to 60% after 3–5 days following a single treatment. However, fluorescence-activated cell sorting (FACS) analysis of tdTomato^+^ epdidermal cells treated with this method revealed fluorescence in only 2% of cells (Wu et al., 2017). Additionally, the increased epidermal-dermal adhesion of the treated mice was not observed after 5 days, highlighting the unknown timeline on the permenance of these gene edits. Furthermore, the potential off-target effects of the RNPs when delivered through electroporation were not analyzed and any potential *in vivo* application will require a more thorough understanding of their RNPs immunogenicity. Though the success of this electroporation technique shows promise for CRISPR/Cas9 gene editing for *in vivo* applications in murine models, considerable limitations including the use of costly specialized instrumentation, pain, collateral damage to the area, and poor understanding of off-target effects must be considered and addressed before it can be deemed safe and effective for humans and appropriate for clinical use. The applied high voltages can cause irreversible changes to the membrane physiology that can adversely affect treated cells. To circumvent the impracticalities of using high voltages for *ex vivo* gene delivery, other methods of cellular poration are under development that preserve the appealing aspects of electroporation, including its scalability and ease of use.

### Acoustoporation

Acoustoporation and sonoporation devices, which utilize ultrasound to induce pore formation in cellular membranes, have also been shown to facilitate the delivery of gene editing cargo. By inducing acoustic waves in a liquid medium, gas-filled microbubbles physically oscillate, often bursting at high pressure and allowing membrane perforation by macromolecules (Helfield et al., 2016). Oscillation facilitated by the negative and positive phases of the incident ultrasound pulses cause alternating expansion and shrinkage of microbubbles, inducing shock waves that disrupt the plasma membrane. At low pressures, the microbubbles undergo stable cavitation, where the magnitude of their oscillating size is inversely proportional to the localized acoustic pressure and disruption to the cell membrane, whereas at high pressures, bubbles undergo inertial cavitation, resulting in large, asymmetric bubble size oscillation and the formation of penetrating liquid jets, as illustrated in Figure 3B. The efficiency of sonoporation is dependent on cellular properties as well as acoustic excitation and microbubble parameters, the understanding of which can increase efficiency and controllability of the system (Tu and Yu, 2022). One such example where acoustoporation was applied to transfect pDNA encoding Cas9 and gRNA to human endometrial cancer (HEC)-1A cells is the work of Cai et al. (2019), who reported a 57% decrease of mRNA expression from the target knockout c-erbB2.

Acoustoporation can be used without the aid of microbubbles, also known as ultrasound contrast-agent microbubbles (CA). These are known to enhance transient poration of cell membranes and are being explored for their *in vitro* and *in vivo* uses, and for their potential clinical applications in gene therapy and drug delivery (Carugo et al., 2011). However, at high pressure CAs can collapse and have the potential to cause capillary rupture and endothelial cell damage *in vivo* and cell rupture *ex vivo* and may reduce the effectiveness of delivered cargoes (Rich et al., 2022). Carugo et al. (2011) demonstrated acoustoporation of cardiac myoblasts in the absence of CAs using a cost-effective ultrasound-microfluidic device, which allowed for control over the position of the cells and the strength of the acoustofluidic forces. Results showed intracellular delivery of pharmaceutical agents (e.g., doxorubicin, luteolin, and apigenin) as well as the transmembrane transfer of fluorescent probes CMFDA and FITC-dextran. Additionally, it was found that cellular uptake of the pharmaceutical agents through acoustoporation in the absence of CAs increases cell cytotoxicity (Carugo et al., 2011). Similarly, Ankrett et al. (2013) investigated the effects of ultrasound-related stimuli without the use of CAs exposing H9c2 cardiac myoblasts to different ultrasonic fields within a glass micro-capillary (Ankrett et al., 2013). The microfluidic device was comprised of a square glass capillary coupled to a piezoelectric transducer (PZT) transducer that was mounted to a glass platform. An optimal injection flow rate of 2.6 ml/h allowed for a high viability of approximately 95% to be maintained (Ankrett et al., 2013). Surface acoustic waves create transient pores in cell membranes and enhance molecular uptake by causing strong streaming in the extracellular microenvironment, also without the use of microbubbles. For example, surface acoustic waves were used in the transfection of a small interfering RNA (siRNA)-liposome complex in HeLa cells and reported 40% transfection efficiency (Ramesan et al., 2018). In addition, hypersonic poration was generated using a nanoelectromechanical resonator to create 200 nm sized pores (Zhang et al., 2017b), and high-frequency bulk acoustic waves at 150 MHz frequency delivered CRISPR plasmid to HeLa and HEK 293 cells with efficiency of up to 40%, depending on size and concentration of the plasmid (Yoon et al., 2017). While typically reserved for *in vitro* applications, acoustoporation as a method of gene editing cargo delivery can in principle work *via* direct navigation by ultrasonic waves through microvasculature to a target tissue. This capability has the potential to offer a noninvasive, image-guided delivery method for the selective release and uptake of the cargo, though it will require extensive experimentation and planning before becoming an accepted method of *in vivo* gene editing (Helfield et al., 2016).

When selecting a delivery vehicle, the viability of cells must be considered. Viability can be effectively optimized in devices which combine the use of ultrasound and microfluidic channels. Such acoustofluidic devices with the potential to create rapidly processing, point-of-care devices for bedside use can be tuned for high viability by adjusting both input voltage as well as flow rate (Belling et al., 2020). Towards this goal, a significant enhancement in the delivery of biomolecules to T cells has been achieved using a 3D printed acoustic device to deliver the fluorescent molecule calcein to human T cells (Centner et al., 2021). Issues of toxicity, cost and throughout have also been addressed *via* the development of an acoustofluidic sonoporation platform (Belling et al., 2020). The effectiveness of this device was demonstrated in the delivery of plasmids to primary human T lymphocytes and clinically relevant cell lines such as peripheral blood mononuclear cells, and CD34^+^ hematopoietic stem and progenitor cells. Acoustofluidic treatment has further been shown to be scalable, achieving throughputs of up to 200,000 cells/min by passing cells through a custom build system over a piezoelectric transducer that is scalable for future CRISPR/Ca9 clinical applications (Belling et al., 2020). Confocal imaging of treated cells revealed the presence of a fluorescent signal attributed to Cy3-labeled DNA at the cell membrane, cytosol, and nucleus for acoustic-treated cells, confirming the successful delivery of cargo using this high throughput method. Aghaamoo et al. (2022) also developed a microfluidic platform to deliver plasmid DNA and sgRNA into cells termed Acousto-Electric Shear Orbiting Poration (AESOP). The platform uses a high-throughput intracellular delivery method that relies on arrays of micro vortices formed by lateral cavity acoustic transducers (LCATs), which trap the cells and induce a controlled mechanical shear and electric field to facilitate the uniform poration on the membrane of a large number of cells simultaneously. AESOP demonstrated the uniform and precise transfection of a wide range of cargoes, including eGFP plasmid (6.1 kbp) and CRISPR/Cas9-mediated gene knockout using a 9.3 kbp plasmid DNA encoding Cas9 protein and sgRNA with viabilities over 80% in both suspension and adherent cell types. As a result, these technologies have the potential to be engineered for a wide variety of therapeutic applications which require large cargoes (Yoon et al., 2017; Wu et al., 2018; Castle et al., 2020).

In addition to cell poration and cargo delivery, acoustofluidic tweezers (a form of acoustophoresis) enable the separation of microparticles and cells while facilitating the controlled and targeted delivery of genetic materials into the cytoplasm (Wu et al., 2018). This intracellular delivery technique has been used to transfect HEK 293 and HeLa cells with DNA plasmid and/or mRNA cargoes to achieve editing at single cell resolution using CRISPR/Cas9 (Yoon et al., 2017). While the use of acoustofluidic tweezers to deliver CRISPR/Cas9 is still limited to *in vitro* studies, acoustoporation has been applied clinically in the delivery of chemotherapeutic agents (Castle et al., 2020). A Phase I clinical trial showed that performing acoustoporation in combination with the chemotherapeutic agent gemcitabine on patients with pancreatic ductal adenocarcinoma demonstrated an increase in the median overall survival from 8.9 to 17.6 months in comparison to 63 historical controls and resulted in no additional adverse effects. The work is now moving forward to a larger Phase II clinical trial (Castle et al., 2020). While these preliminary efforts show great promise, there are still several obstacles that need to be optimized for acoustoporation to increase its clinical potential.

## Particle-based delivery

**FIGURE 4:**
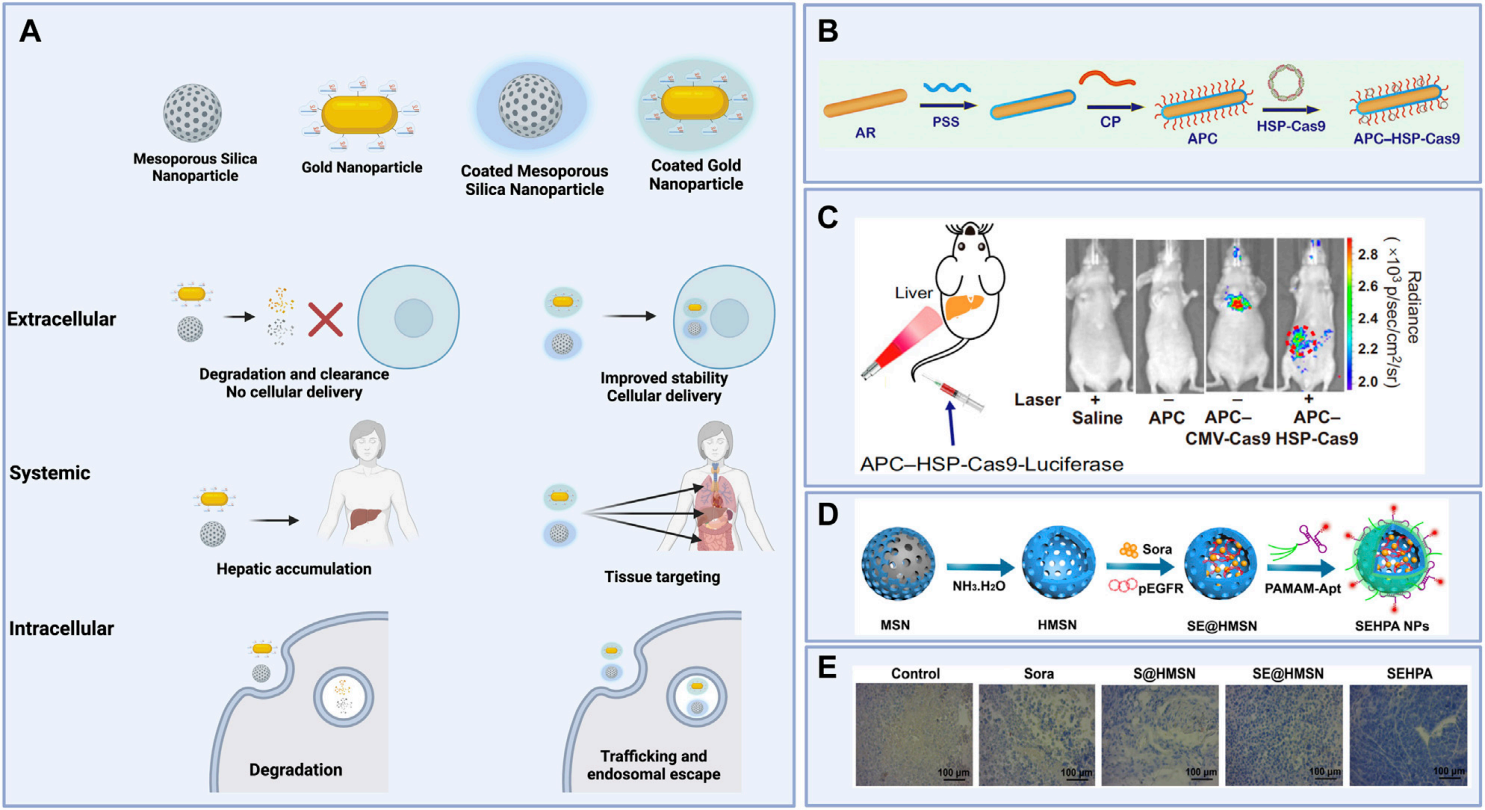
**(A)** Schematic illustrating extracellular, systemic, and intracellular effects of organic coatings for inorganic nanoparticle cores carrying gene editing cargoes. **(B)** Polystyrene sulfonate (PSS)- and β-cyclodextrin-polyethyleneimine (CD-PEI)-coated gold nanorods (APCs) then complexed to a Cas9 plasmid construct with a heat-inducible promoter for spatiotemporal control of gene editing induced by laser irradiation. **(C)** Luciferase expression after laser irradiation of the liver in mice treated with APCs carrying Cas9 plasmid constructs without a heat-inducible promoter (APC-CMV-Cas9) versus APCs carrying Cas9 plasmid constructs with a heat-inducible promoter (APC-HSP-Cas9). **(D)** Synthesis of hollow mesoporous silica nanoparticles (HMSNs) loaded with Sorafenib and Cas9 plasmids targeting the EGFR locus, then coated with poly (amidoamine) (PAMAM) and an anti-EpCAM DNA aptamer to form coated nanoparticles (SEHPA NPs). **(E)** Immunohistochemistry staining shows reduced EGFR expression in tumor tissue treated with SEHPA NPs compared to the controls and to uncoated HMSNs. Figures 4B,C reused from Chen et al., 2020 with permission from Proceedings of the National Academy of Sciences of the United States of America. Figures 4D,E reprinted with permission from (Zhang et al., 2020) Copyright 2020 American Chemical Society. Schematics created using BioRender.

While promising for *in vitro* and *ex vivo* applications, the physical methods of delivery mentioned above do not offer feasible solutions to *in vivo* gene editing because they must be performed external to the patient. Particle carriers are better suited to such applications as they can be administered systemically. Viral particles have high transfection efficiencies, but have limited packaging capacities, are prone to immune activation and off-target effects, and may undergo recombination events, which can produce replication-competent viruses (Ashok et al., 2021). Synthetic carriers for CRISPR/Cas9 cargoes may offer a solution to these challenges. These synthetic carriers may be polymeric, lipid-based, or inorganic, and are appealing due to their tunability towards specific applications (Figure 4).

### Polymeric and supramolecular nanocarriers

Cationic polymers such as chitosan and poly (ethyleneimine) (PEI) have long been used for the delivery of biomolecules, but confer particular challenges with delivery inefficiencies, problems with solubility, and toxicity (Ping et al., 2011; Ashok et al., 2021). These issues have been addressed, in part, by combining multiple constituents to obtain particles with superior efficacy to any one polymeric component alone. For example, Ma et al. (2021) compared genomic integration via delivery of plasmid encoding Cas9 using either particles comprised of polydopamine (PDA) and PEI alone, or functionalized with hyaluronic acid (HA), which is thought to target carbohydrate-specific endocytotic receptors, and dexamethasone (DEX), which acts as a nuclear localization signal, and reported higher rates of integration with functionalized particles.

Further improvements to nanoparticle efficacy have been pursued through the development of custom-designed polymers as well as modifications to well-established polymers to improve their efficacy. Specifically, adamantane is used to modify nanoparticle components such as dendrimers, as its lipophilic properties support stable incorporation of functional components including transactivator of transcription (TAT) sequences and polyethylene glycol (PEG) moieties into self-assembled structures (Štimac et al., 2017). Similarly, biocompatible molecules are often grafted to PEI to improve its efficacy as a delivery agent. For example, *in vitro* gene editing efficiencies achieved *via* PEI-based nanoparticle delivery of CRISPR/Cas9 reagents are improved when PEI components are functionalized with HA or both HA and mannose (Francis et al., 2022). This enhancement is thought to be a result of the ability of these functionalities to target carbohydrate-specific endocytotic receptors on certain cell types (Francis et al., 2022). Further, modifying PEI with β-cyclodextrin to form CD-PEI has become common practice to generate nanoparticles with similar packaging and endosomal escape efficiency to PEI but much lower toxicity and, as a result, better transfection efficiencies (Ping et al., 2011). These grafting approaches are commonly utilized and effective for generating supramolecular polymeric nanocarriers. These modified compounds are leveraged as components of supramolecular nanoparticles (SMNPs), comprised of optimized ratios of adamantane-grafted polyamidoamine dendrimer (Ad-PAMAM), adamantane-grafted poly (ethylene glycol) (Ad-PEG), and CD-PEI, for the delivery of CRISPR/Cas9 reagents (Chou et al., 2020). In one study, SMNPs encapsulating plasmid encoding Cas9 and a guide targeting a safe-harbor locus and, separately, a plasmid encoding GFP plus a functional donor induced the correction of RS1 *in vitro* following intravitreal injection in a mouse model (Chou et al., 2020). Nanowire-grafted SMNPs were optimized to package and deliver RNPs as well, inducing successful disruption of the dystrophin gene and knock-in of the HBB gene (Yang et al., 2020; Ban et al., 2021). Similarly, Wan et al. (2020) used disulfide-bridged biguanidyl adamantane (Ad-SS-GD) with CD-PEI as components for supramolecular assembly for the packaging and delivery of Cas9 RNPs and successfully induced nearly 16% editing in SW-280 cells. Further decorating these Ad-SS-GD/CD-PEI nanoparticles with HA enabled *in vivo* tumor-specific editing at the KRAS gene in mice *via* intravenous (IV) administration (Wan et al., 2020).

**FIGURE 5:**
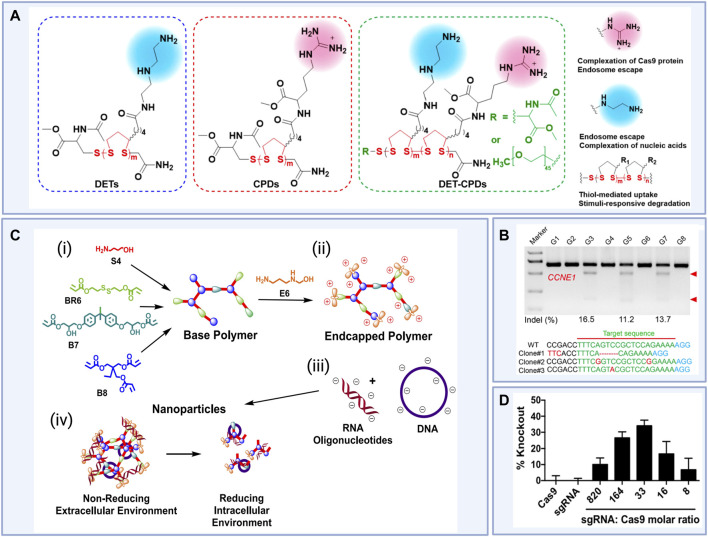
(i) Rational design of novel diethylenetriamine cell-penetrating poly (disulfide)s (DET-CPDs) for use in nanoparticle-based gene delivery and (ii) *in vivo* indel frequencies detected by the T7E1 assay from murine liver tissue and representative Sanger sequencing results of T-A cloning from liver tissue after treatments (Clone 1, DET-CPD-12/CMV-Cas9-sgCCNE1; Clone 2, DET-CPD-12/Cas9 mRNA sgCCNE1; Clone 3, DET-CPD-12/Cas9 RNP-sgCCNE1) where DET-CPD-12 was the top-performing poly (disulfide) DET-CPD and G3, G5, and G7 represent Clones 1, 2, and 3 respectively. G1 and G2 represent phosphate buffered saline (PBS) controls and G4, G6, and G8 represent mock gRNA treatments. (iii) Design of R6,7,8_64, a novel reducible branched poly (amino ester) (rPBAE) for use in nanoparticle-based gene delivery and (iv) percent green fluorescent protein (GFP) knockout in HEK 293Ts stably expressing GFP induced For example, *in vitro* gene editing efficiencies achieved via R6,7,8_64 particle-mediated delivery of Cas9 plasmid constructs and anti-GFP sgRNA at a variety of sgRNA to Cas9 molar ratios compared to R6,7,8_64 particle-mediated delivery of Cas9 or sgRNA alone. Figure 5 (i) and (ii) reused from Guo et al., 2021 with permission from American Chemical Society. License CC BY-NC-ND 4.0 https://creativecommons.org/licenses/by-nc-nd/4.0/. Figure 5 (iii) and (iv) reprinted with permission from (Rui et al., 2019b) Copyright 2019 American Chemical Society. Schematics created using BioRender.

Other attempts at addressing the cytotoxicity issues that often accompany the use of polymeric nanoparticles include the use of materials that are degradable under physiological conditions. These materials generally include reducible functional groups, including disulfides, esters, and aminoesters. For example, Guo et al. (2021) rationally designed a series of poly (disulfide)s generated to form bioreducible materials with high cellular uptake efficiency, then explored their potential for nanoparticle delivery of Cas9-encoding plasmid, Cas9-encoding mRNA, sgRNA, and RNP complexes (Guo et al., 2021). The top-performing poly (disulfide) induced high rates of *in vitro* indel frequencies for all types of cargo, and these results were corroborated by slightly lower but still remarkable levels of indel frequencies in the liver after IV administration of the nanoparticles (Figure 5). This sort of rational design and experimentation is common in the development of new materials for nanoparticle delivery. For example, similar extensive investigations into poly (aminoesters) (PBAEs) as a key nanoparticle component for delivery of CRISPR/Cas9 genome editing reagents have been investigated (Rui et al., 2019a; Rui et al., 2019b; Rui et al., 2020). Indeed, 40% gene knockout was demonstrated *in vitro* by codelivery of plasmid encoding Cas9 as well as guide RNA targeting a fluorescent reporter gene *via* reducible branched ester-amine quadpolymer (rBEAQ)-based nanoparticles (Figure 5).

Custom polymers under investigation are intended to resolve the challenge of packaging Cas9 RNPs by enabling covalent functionalization to amines on the endonuclease surface. For example, the work by Rui et al. (2019b) on PBAEs was extended to design carboxylated branched PBAEs nanoparticle carriers that are capable of RNP encapsulation (Rui et al., 2019a). These polymers are distinguished by their capability to react with the amines present at the surface of RNPs. Delivery of these particles induced over 75% gene knockout and 4% knock-in *in vitro*, and intracranial administration induced gene editing *in vivo* in mice bearing glioma tumors. In a similar approach, rather than modifying PAMAM with adamantane, Liu et al. (2019a) reports the use of boronic acid-functionalized PAMAM to successfully induce high indel rates *in vitro*. This moiety is intended to provide a functional group that enables conjugation with the amines present at the RNP surface.

Finally, polymers are appealing for particle-mediated CRISPR/Cas9 delivery because their broad biochemical properties may not only facilitate delivery and packaging but confer some therapeutic benefit on their own. As a result, indirect approaches that synergistically complement the *in vivo* editing efficiencies of particle-based delivery, particularly for tumor therapies, include use of polymers sourced from non-CRISPR gene disrupting agents. Specifically, Zhang et al. (2021a) reports a cationic platinum (Pt (IV))-backboned polymer chain, derived from cisplatin, delivering a plasmid encoding both Cas9 and a guide sequence induced 32.2% gene disruption *in vitro* and 21.3% of tumor tissues *in vivo*.

### Lipid nanocarriers

While polymeric nanoparticles for CRISPR/Cas9 delivery have undergone significant advancements, they present with toxicity issues and tend to lack targeting specificity. Lipid nanoparticles (LNPs) offer a platform to overcome many of these challenges. Similarly to unmodified cationic polymers, cationic lipid constituents tend to confer issues with toxicity. This challenge has been largely overcome with the use of ionizable lipids that preserve electrostatic interactions with anionic cargoes. In addition, straightforward lipid self-assembly may be leveraged to be scalable and their modular composition has enabled some amenability to targeting specific tissues, even *via* systemic administration (Liu et al., 2021). For these reasons, LNPs are commonly used nanocarriers for *in vivo* delivery of nucleic acids and in particular, CRISPR/Cas9 therapies. Commercially available lipid-based transfection reagents such as Lipofectamine™ have long been used to deliver biomolecules to cells *in vitro* (Zangi et al., 2013; Lou et al., 2020), and these reagents have therefore been used as a standard against which next-generation LNP formulations are compared. This benchmarking has enabled the continual improvement and optimization of LNP formulations and novel ionizable lipids and lipid-like materials. Specifically, DLin-MC3-DMA has largely replaced commercial lipofection reagents as the preferred material for nucleic acid delivery and, in particular, siRNA (Jayaraman et al., 2012; Hou et al., 2021). This synthetic lipid not only serves as a standard delivery agent *in vitro* but has been used for *in vivo* delivery as well. For example, Onpattro^®^, comprising in key part Dlin-MC3-DMA, is the first siRNA drug approved by the United States Food and Drug Administration (FDA) (Akinc et al., 2019). While this lipid is considered the most reliable commercially available lipid carrier for nucleic acids, it is not optimized for mRNA or RNP delivery, and is limited in efficacy and tolerability by its non-degradability. As Dlin-MC3-DMA does not represent a perfect lipid carrier for CRISPR/Cas9-based cargoes, efforts have been made to engineer biodegradable lipids with improved pharmacokinetics (Maier et al., 2013). This research has resulted in clinically suitable mRNA delivery materials, including the lipid-like compounds SM-102 and ALC-0315 used in the mRNA-1273 and BNT162b vaccines against COVID-19 (Hassett et al., 2019; Hou et al., 2021).

Several groups have generated combinatorial libraries of synthetic lipids and lipid-like materials using rational design techniques similar to those outlined in Figure 5. These libraries are summarized in Table 1.

**TABLE 1:** Summary of novel lipids and lipidoids systematically designed and screened for their capabilities to deliver gene editing cargoes to cells.

Hits	Group	# Lipids screened	Linker backbone	Lipid chain	Head group	Target	Cargo	Findings
NTA-EC16 performed best for delivery of and editing induced by RNPs	(Li et al., 2018b; Li et al., 2022)	3	Alkyl or ester	Saturated 12-carbon chains with hydroxyl, ether, or disulfide group at C2	Nitriloacetic acid (NTA)	HEK 293	RNP	Tail structure impacts transfection efficiency and toxicity profile
306-O12B	Qiu et al. (2021)	24	Ester	Saturated 12-carbon chains with disulfide group at C2	Tertiary amine head	tdTom mouse model	Cas9 mRNA + gRNA	306-012B outperformed DLin-MC3-DMA
80-O17Se, 81-O17Se, and 400-O17Se performed best for editing induced by RNPs	Li et al. (2022)	51	Ester	Saturated chalcogen-containing alkyl chains (containing O, S, or Se)	17 commercially available head groups with 1, 2, or 3 ionizable amines	HEK 293	RNP	O17Se tails, head groups containing 1 or 2 N atoms and at least one tertiary amine most likely to be effective
83-O14B, 4-O14B, and 6-O14B	Wang et al. (2016)	12	Ester	Saturated 12-, 14-, 16, or 18-carbon alkyl chain containing disulfide bond	Primary or secondary amines	EGFP-expressing HEK 293	RNP	14-, 16-, and 18-carbon chains most efficacious
5A2-SC8	Liu et al. (2021)	572 (termed iPhos lipids)	Phosphate	Saturated carbon tails	28 primary, secondary, and tertiary amines	tdTom mouse model	Cas9 mRNA + gRNA	Most efficacious iPhos lipids contained one ionizable amine, one phosphate group, and three hydrophobic tails. Chain length on amine impacted efficacy (8–10 carbons optimal); chain length on phosphate group influenced organ selectivity (shorter chains directed towards liver, longer chains directed towards spleen)

From these libraries, several novel ionizable lipids have been shown to deliver CRISPR/Cas9 reagents effectively. Several groups have demonstrated *in vitro* and *in vivo* gene editing using fluorescent reporter models, and some have extended their investigation to clinically-relevant genes, such as those overexpressed in tumor cells. These next generation lipids include branched-tail and bioreducible lipids to improve encapsulation, endosomal escape, editing efficiency and reduce toxicity when delivering Cas9-encoding mRNA as compared to DLin-DMA-MC3. The improvements outlined above demonstrate the advancing potential of LNPs for CRISPR/Cas9 therapies both *in vitro* and *in vivo* (Qiu et al., 2021). Most of these lipids, however, have not resolved the challenge of preferential particle accumulation in the liver, and other tissues remain difficult to target *via* systemic administration. This is advantageous only when the target tissue is the liver, and indeed there have been clinical examples of gene editing in the liver. Specifically, Gillmore et al. (2021) present clinical data showing therapeutic potential of an LNP composition containing the proprietary lipid NTLA-2001 to induce editing in the *Ttr* gene for patients with hereditary transthyretin amyloidosis (ATTR). Early clinical studies in humans have shown reductions from baseline serum levels of TTR protein up to 96%.

Cheng et al. (2020) has taken combinatorial synthesis a step further and developed a combinatorial methodology of LNP composition to determine the impact of LNP constituents and their ratios on organ selectivity. With this methodology, LNP constituents were demonstrated to behave as selective organ targeting (SORT) molecules that influence the tissue-localization of gene editing after intravenous administration. When top-performing iPhos lipid (5A2-SC8) and dioleoyl-3-trimethylammonium propane (DOTAP) were used as model constituents, tissue-specific protein expression was tunable according to the ratio of the SORT molecule (in this case, DOTAP) in the LNP composition. Specifically, LNPs carrying Cas9 mRNA with 20% molar ratio of DOTAP induced gene editing exclusively in the liver, whereas LNPs with 50% DOTAP induced protein expression exclusively in the lung. The corresponding shift in editing distribution from liver to lung transgressed through the spleen at mid-range DOTAP percentages (Cheng et al., 2020). A similar trend was noted for the delivery of RNPs. This design strategy has larger implications for the clinical feasibility of systemically-administered nanoparticle-based gene therapy. Until this work, systemic administration for virtually all types of nanocarriers has resulted in preferential accumulation in the liver and limited application for targeting other tissues. Exceptions to this trend include LNPs functionalized with tumor-targeting moieties to enhance gene editing specifically in tumors, rather than other tissues. For example, a cationic lipid with a phenylboronic acid (PBA) moiety has been used to improve specificity of interaction of LNPs with the upregulated sialic acid expression at the surface of cancer cells (Tang et al., 2019). This lipid, called PBA-BADP, was used to deliver Cas9 mRNA and sgRNA targeting the GFP gene in GFP-expressing HeLa cells. PBA-functionalized LNPs induced GFP knockout of nearly 50%, as compared to less than 30% knockout induced by non-PBA functionalized LNPs (Tang et al., 2019). Tumor cells may alternatively be directly targeted using LNPs tagged with antibodies. Selective uptake of antibody-targeting LNPs administered intraperitoneally by disseminated tumors has demonstrated ∼80% gene editing *in vivo* (Finn et al., 2018). In an alternative approach, Liu et al. (2019b) report successful delivery using a bioreducible lipid/Cas9 mRNA nanoparticle, BAMEA-O16B (C_56_H_111_N_3_O_6_S_6_) in the treatment of human cervical cancer cells. The efficient delivery revealed genome editing present after just 24 h and knock-out of GFP with up to 90% efficiency.

While these advancements have largely addressed issues with toxicity, encapsulation, and transfection efficiency for negatively-charged mRNA, the instability of RNPs in the acidic environment required for electrostatic complexation with ionizable lipid components presents a barrier to efficient complexation with cationic lipids to form LNPs (Walther et al., 2022). Notably, there has been limited success in delivering RNPs using LNP platforms, with a few exceptions, including those reported in Table 1. In addition, Walther et al. (2022) report the combination of a pH-neutral buffer and permanently cationic lipid components enabled successful encapsulation of RNPs with 19.2% HDR induced *in vitro* by RNPs encapsulated within LNPs composed of C12-200, 1,2-dioleoyl-sn-glycero-3-phosphoethanolamine (DOPE), cholesterol, poly (ethylene glycol)-1,2-dimyristoyl-rac-glycerol (PEG-DMG), and DOTAP (Walther et al., 2022). This formulation method, along with those featured in Table 1, exemplify the ability to overcome challenges initially identified in the complexation of LNPs and provide a foundation upon which further strides can be made towards the broader clinical translation of LNP vehicles for gene therapies.

### Inorganic nanocarriers

Despite the advances outlined above, challenges remain in designing nanocarriers that accommodate hard-to-package cargoes like Cas9 RNPs or that provide the stimulus-responsive control of tissue targeting, intracellular trafficking, and cargo release. Inorganic nanocarriers such as gold nanoparticles (AuNPs), mesoporous silica nanoparticles (MSNs), and metal-organic frameworks (MOFs) are appealing due to their versatility and ease of chemical functionalization. These attributes enable creative strategies to address these barriers to delivery. However, each of these types of particles presents with preferential accumulation in the liver and/or biocompatibility issues that limit their efficacy for systemically administered CRISPR/Cas9 therapies. Recent work in inorganic nanoparticle delivery has been largely focused on developing core-shell structures that incorporate organic components to circumvent these issues, and engineered stimulus-responsiveness or tissue-targeting strategies to control the localization of gene editing cargo (Figure 4). While MOFs are under rapid and intense investigation, this review will focus on AuNPs and MSNs due to their higher scalability.

#### Gold nanoparticles

The plasmonic properties of AuNPs confer a portfolio of targeting and release capabilities and are frequently leveraged in the design of complex nanocarriers for gene editing reagents. Mout et al. (2017) synthesized arginine-functionalized AuNPs, effectively generating positively-charged particles (Mout et al., 2017). By then tagging Cas9 with glutamate, forming a negatively-charged patch on the otherwise cationic protein, the group enabled complexation between Cas9 and the AuNPs to form nanoassemblies. The resulting nanoassemblies exhibited 90% delivery efficiency and indel efficiency in HeLa cells of 29–30% (Mout et al., 2017). Building on this work, Ray et al. (2018) demonstrated gene editing to knockout macrophage signal regulatory protein-α (SIRP-α) in macrophages to promote phagocytosis of cancer cells *in vitro* (Ray et al., 2018). Further, colloidal AuNPs have been developed for the codelivery of guide RNA and Cas9 with or without an ssODN. Guide RNAs were attached to the AuNP surface *via* oligo ethylene glycol (OEG) spacers with terminal thiol linkers, and Cas9 proteins were subsequently complexed to the tethered guide RNAs (Shahbazi et al., 2019). To enable further electrostatic complexation to an ssODN, the AuNP assembly was further coated with branched PEI. Treatment of hematopoietic stem/progenitor cells (HSPCs) with these nanoassemblies resulted in 17.6% total editing with 13.4% HDR at the CCR5 locus with minimal toxicity (Shahbazi et al., 2019).

While these approaches work well *in vitro*, there are very few examples of AuNPs being applied for delivery and diagnostic applications in clinical trials as reviewed recently by Singh et al. (2018), and, to date no examples of the clinical translation of CRISPR-based gene editing using AuNP-based approaches have been reported (Singh et al., 2018). Common approaches to overcoming additional barriers to clinical *in vivo* stability, biocompatibility, and efficacy of AuNPs include the utilization of polymers or lipids to coat the AuNP, protect the cargo, and aid in endosomal escape. Lee et al. (2017) used an endosomal disruptive polymer, PAsp(DET), as a coating to accomplish these objectives. In this example, AuNPs were conjugated with glutathione linkers to DNA, which enabled the electrostatic complexation of Cas9 RNP and ssODN cargoes. After endosomal release promoted by the buffering effect of PAsp(DET), cytoplasmic conditions induced cleaving of the glutathione linker to release the cargo and enable gene editing, including HDR-mediated insertion of a ssODN template designed to convert blue fluorescent protein (BFP) to GFP in a BFP-expressing HEK reporter cell line (Lee et al., 2017). Lipids may also be used as coatings for AuNPs. Wang et al. (2018b) developed AuNPs functionalized with cationic TAT peptides to enable complexation with plasmid encoding Cas9 and gRNA-Plk-1 to form TAT peptide-modified gold nanoparticles (ACPs) (Wang et al. (2018b). These were subsequently encapsulated by a lipid composition including DOTAP, DOPE, cholesterol, and PEG anchored to 1,2-Distearoyl-sn-glycero-3-phosphoethanolamine (PEG2000-DSPE) to form lipid encapsulated-ACPs (LACPs). Apoptosis was induced *in vitro*, and *in vivo* mouse models showed tumor inhibition induced by treatment with LACPs. Compared to a PBS control, tumors injected with LACPs were reduced in size by 42% at the time of sacrifice.

The LACPs outlined above induced much higher levels of gene editing and tumor inhibition when irradiated with near infrared (NIR) light, leveraging the plasmonic properties of AuNPs for the stimulus-responsive release of cargo, and importantly presenting a platform for controlling the location of gene editing even after systemic administration (Wang et al., 2018b). Chen et al. (2020) synthesized gold nanorods with a coating of polystyrene sulfonate (PSS) beneath an outer coating of β-cyclodextrin-polyethyleneimine (CD-PEI), which could then be complexed to a Cas9-encoding plasmid including a heat-inducible promoter (Chen et al., 2020). These particles, dubbed APCs, were used as an optogenetic switch for gene editing (Chen et al., 2020). Irradiating APC-treated tissue in the NIR induces plasmonic heating, thereby activating transcription of the plasmid. The group found that Cas9 expression was inducible *via* Western blot analysis as well as flow cytometry quantifying GFP knockout in GFP-expressing HEK 293Ts. *In vivo* mouse models showed peritumoral injection of APCs targeting the Plk1 locus to induce significant on-target mutation and reduction in tumor size only when the tumors were irradiated with NIR (Chen et al., 2020). This system enabled precise control of editing only at the sites of interest.

#### Silica nanoparticles

Silica nanoparticles are of interest for CRISPR/Cas9 gene therapies due to their tunability to respond to stimuli and their porosity, which enables high encapsulation efficiencies and co-delivery of small-molecule drugs (Xu et al., 2021). However, like AuNPs, *in vivo* administration of mesoporous silica nanoparticles (MSNs) presents challenges with stability of both carrier and cargo and poor control over release kinetics (Xu et al., 2021). To resolve these issues, more complex structures are being investigated for improving MSN efficacy. To date, while clinical trials are underway for MSN-based drug delivery, diagnostic, and theranostic applications as reviewed recently (Janjua et al., 2021), no CRISPR-based MSN therapies have reached this stage of clinical investigation.

Similar to AuNPs, lipids and polymers are frequently used as coatings for mesoporous silica for the delivery of Cas9-encoding plasmids and RNPs. Noureddine et al. (2020) reports MSNs coated in DOTAP, DOPE, DSPE-PEG2000, and cholesterol for the delivery of RNPs to induce 10% gene editing both *in vitro* and locally *in vivo* after intracranial administration in mice (Noureddine et al., 2020). Lipids and polymers including PAMAM (Zhang et al., 2020), PDDA (Xu et al., 2021), and PEG (Wang et al., 2021) have been used to coat MSNs loaded with both a small molecule drug and RNPs. Liu used a similar lipid-coated MSN system to co-deliver a small molecule drug and RNPs (Liu et al., 2020). Tissue-targeting moieties may easily be conjugated to some of these coatings, including hepatocyte-targeting *N*-acetylgalactosamine (GalNAc) (Wang et al., 2021).

For applications where target cells are not hepatocytes, chemical functionalization is one approach leveraged to avoid preferential accumulation in the liver. Liu et al. (2019b) reported coating RNP-loaded MSNs with PBA-modified PEI for tumor-targeting, then complexing the resulting polyplex with 2,3-dimethylmaleic anhydride (DMMA)-modified poly (ethylene glycol)-*b*-polylysine (mPEG_113_-*b*-PLys_100_/DMMA) to protect the cargo from degradation. With this system, *in vitro* and *in vivo* tumor-targeted gene editing was successfully induced. Alternatively, MSNs may be engineered to respond to stimuli for spatial and temporal control of gene editing after systemic administration. Silica-based up-conversion nanoparticles (UCNPs) are frequently leveraged to deliver gene editing reagents in a stimulus-responsive manner. Pan et al. (2019) synthesized lanthanide-doped UCNPs coated in SiO_2_ functionalized with UV-photocleavable 4-(hydroxymethyl)-3-nitrobenzoic acid (ONA) linkers directly to Cas9 RNPs and subsequently encapsulated within a PEI layer (Pan et al., 2019). The RNPs targeted Plk-1 to investigate the utility of these particles to inhibit tumor growth. These UCNPs upconvert incident biologically safe NIR to ultraviolet (UV) radiation, which subsequently results in cleavage of the linker to release the RNP from the UCNP (Pan et al., 2019). The group confirmed gene editing by knocking out GFP expression in GFP-transduced KB cells, and induced apoptosis by knocking out the Plk-1 gene in A549 cells. These results were corroborated in *vivo* mouse models, which displayed indels induced after intratumoral UCNP administration in tumor tissue and reduced tumor size compared to controls (Pan et al., 2019).

## Summary and future prospects

This review serves to summarize current approaches to optimizing the CRISPR/Cas9 system in both its cargo and methods of delivery, and the challenges those approaches aim to address. Recent advances in biotechnology developed for the safe and effective utility of CRISPR/Cas9 payloads have improved the outlook for clinical applications of gene editing. Modifications made to gene editing biomolecules to reduce their inherent toxicity and the risk of off-target effects operate synergistically with methods of delivery that have undergone significant technological improvements since their introduction.

While broad clinical use of CRISPR/Cas9-based therapies is still on the horizon, many of the safety and scalability challenges that have formerly served as obstacles to clinical translation are actively being addressed. *Ex vivo* methods of gene delivery have undergone significant advancements and have demonstrated clinical applicability and effectiveness. Specifically, cell therapies involving CRISPR-based genetic modification of CAR T cells and hematopoietic stem cells are under clinical development and investigational trials for treating cancer, β-thalassemia, sickle cell anemia, HIV, and refractory B cell malignancies are underway (Hirakawa et al., 2020). Despite this rapid progress, barriers to efficient *in vivo* systemic delivery of CRISPR/Cas9 reagents and induction of therapeutic levels of gene editing in tissues of interest have not been completely overcome. CRISPR/Cas9 therapies undergoing interventional trials for direct use *in vivo* are extremely limited, including only an AAV-based application for Leber congenital amaurosis and a topical treatment for human papillomavirus (HPV)-related malignancies (Hirakawa et al., 2020). Creative and novel approaches to administering *in vivo* CRISPR/Cas9 therapies may be required to circumnavigate current barriers to systemic administration, achieving appropriate safety profiles, improving fiscal scalability, realizing delivery cargo and disease agnostic solutions that are robust and effective while being compliant with Good Manufacturing Practices remain of high concern to ensure equitable availability of emerging gene therapeutic interventions. While tools to improve gene editing are becoming standard practice in research laboratories, to achieve clinical translational goals for CRISPR/Cas9 gene therapies it is imperative that scientists in related fields of molecular biology, chemistry, medicine, and engineering continue to collaborate and stay up to date with recent advances. The reviewed literature indicates a fast-paced trajectory for CRISPR/Cas9-based therapeutics and technologies facilitated by multilevel and multifaceted approaches to reliably safe, scalable, and effective intracellular delivery.

## References

[B1] Aghaamoo M. Chen Y.-H. Li X. Garg N. Jiang R. Yun J. T.-H. (2022). High-throughput and dosage-controlled intracellular delivery of large cargos by an acoustic-electric micro-vortices platform. Adv. Sci. 9 (1), 2102021. 10.1002/advs.202102021 PMC8728830 34716688

[B2] Akinc A. Maier M. A. Manoharan M. Fitzgerald K. Jayaraman M. Barros S. (2019). The Onpattro story and the clinical translation of nanomedicines containing nucleic acid-based drugs. Nat. Nanotechnol. 14 (12), 1084–1087. 10.1038/s41565-019-0591-y 31802031

[B3] Alghadban S. Bouchareb A. Hinch R. Hernandez-Pliego P. Biggs D. Preece C. (2020). Electroporation and genetic supply of Cas9 increase the generation efficiency of CRISPR/Cas9 knock-in alleles in C57BL/6J mouse zygotes. Sci. Rep. 10 (1), 17912. 10.1038/s41598-020-74960-7 33087834 PMC7578782

[B4] Ankrett D. N. Carugo D. Lei J. Glynne-Jones P. Townsend P. A. Zhang X. (2013). The effect of ultrasound-related stimuli on cell viability in microfluidic channels. J. nanobiotechnology 11, 20. 10.1186/1477-3155-11-20 23809777 PMC3706218

[B5] Ashok B. Peppas N. A. Wechsler M. E. (2021). Lipid- and polymer-based nanoparticle systems for the delivery of CRISPR/Cas9. J. Drug Deliv. Sci. Technol. 65, 102728. 10.1016/j.jddst.2021.102728 34335878 PMC8318345

[B6] Ban Q. Yang P. Chou S.-J. Qiao L. Xia H. Xue J. (2021). Supramolecular nanosubstrate-mediated delivery for CRISPR/Cas9 gene disruption and deletion. Small 17 (28), 2100546. 10.1002/smll.202100546 PMC8282741 34105245

[B7] Belling J. N. Heidenreich L. K. Tian Z. Mendoza A. M. Chiou T.-T. Gong Y. (2020). Acoustofluidic sonoporation for gene delivery to human hematopoietic stem and progenitor cells. Proc. Natl. Acad. Sci. U. S. A. 117(20), 10976–10982. 10.1073/pnas.1917125117 32358194 PMC7245081

[B8] Bhattacharya D. Marfo C. A. Li D. Lane M. Khokha M. K. (2015). CRISPR/Cas9: An inexpensive, efficient loss of function tool to screen human disease genes in Xenopus. Dev. Biol. 408 (2), 196–204. 10.1016/j.ydbio.2015.11.003 26546975 PMC4684459

[B9] Bower D. M. Prather K. L. (2009). Engineering of bacterial strains and vectors for the production of plasmid DNA. Appl. Microbiol. Biotechnol. 82 (5), 805–813. 10.1007/s00253-009-1889-8 19205691

[B10] Bridgen D. DiTommaso T. Bugge J. Gilbert J. Bernstein H. Sharei A. (2017). “Vector-free genome editing of primary immune cells for cell therapy,” in *Molecular therapy*: Cell press 50 hampshire st, floor 5 (CAMBRIDGE, MA 02139 USA, 23–24.

[B11] Cai J. Huang S. Yi Y. Bao S. (2019). Ultrasound microbubble-mediated CRISPR/Cas9 knockout of C-erbB-2 in HEC-1A cells. J. Int. Med. Res. 47 (5), 2199–2206. 10.1177/0300060519840890 30983484 PMC6567764

[B160] Cao Y. Chen H. Qiu R. Hanna M. Ma E. Hjort M. (2018). Universal intracellular biomolecule delivery with precise dosage control. Sci. adv. 4 (10), eaat8131. 30402539 10.1126/sciadv.aat8131 PMC6209385

[B12] Cao Y. Ma E. Cestellos-Blanco S. Zhang B. Qiu R. Su Y. (2019). Nontoxic nanopore electroporation for effective intracellular delivery of biological macromolecules. Proc. Natl. Acad. Sci. U. S. A. 116 (16), 7899–7904. 10.1073/pnas.1818553116 30923112 PMC6475394

[B13] Carugo D. Ankrett D. N. Glynne-Jones P. Capretto L. Boltryk R. J. Zhang X. (2011). Contrast agent-free sonoporation: The use of an ultrasonic standing wave microfluidic system for the delivery of pharmaceutical agents. Biomicrofluidics 5 (4), 044108–4410815. 10.1063/1.3660352 PMC3364807 22662060

[B14] Castle J. Kotopoulis S. Forsberg F. (2020). Sonoporation for augmenting chemotherapy of pancreatic ductal adenocarcinoma. Methods Mol. Biol. 2059, 191–205. 10.1007/978-1-4939-9798-5_9 31435922 PMC7418147

[B15] Catanese D. J. Jr. Fogg J. M. Schrock D. E. 2nd Gilbert B. E. Zechiedrich L. (2012). Supercoiled Minivector DNA resists shear forces associated with gene therapy delivery. Gene Ther. 19 (1), 94–100. 10.1038/gt.2011.77 21633394 PMC3252587

[B16] Centner C. S. Moore J. T. Baxter M. E. Long Z. T. Miller J. M. Kovatsenko E. S. (2021). Acoustofluidic-mediated molecular delivery to human T cells with a three-dimensional-printed flow chamber. J. Acoust. Soc. Am. 150 (6), 4534–4547. 10.1121/10.0009054 34972278

[B17] Chakrabarty P. Gupta P. Illath K. Kar S. Nagai M. Tseng F.-G. (2021). Microfluidic mechanoporation for cellular delivery and analysis. Mat. Today Bio 13, 100193. 10.1016/j.mtbio.2021.100193 PMC8718663 35005598

[B18] Chen G. Abdeen A. A. Wang Y. Shahi P. K. Robertson S. Xie R. (2019). A biodegradable nanocapsule delivers a Cas9 ribonucleoprotein complex for *in vivo* genome editing. Nat. Nanotechnol. 14 (10), 974–980. 10.1038/s41565-019-0539-2 31501532 PMC6778035

[B19] Chen X. Chen Y. Xin H. Wan T. Ping Y. (2020). Near-infrared optogenetic engineering of photothermal nanoCRISPR for programmable genome editing. Proc. Natl. Acad. Sci. U. S. A. 117 (5), 2395–2405. 10.1073/pnas.1912220117 31941712 PMC7007568

[B20] Cheng Q. Wei T. Farbiak L. Johnson L. T. Dilliard S. A. Siegwart D. J. (2020). Selective organ targeting (SORT) nanoparticles for tissue-specific mRNA delivery and CRISPR–Cas gene editing. Nat. Nanotechnol. 15 (4), 313–320. 10.1038/s41565-020-0669-6 32251383 PMC7735425

[B21] Chou S. J. Yang P. Ban Q. Yang Y. P. Wang M. L. Chien C. S. (2020). Dual supramolecular nanoparticle vectors enable CRISPR/Cas9‐Mediated knockin of retinoschisin 1 gene—a potential nonviral therapeutic solution for X‐linked juvenile retinoschisis. Adv. Sci. (Weinh). 7 (10), 1903432. 10.1002/advs.201903432 32440478 PMC7237855

[B22] Chu V. T. Weber T. Wefers B. Wurst W. Sander S. Rajewsky K. (2015). Increasing the efficiency of homology-directed repair for CRISPR-Cas9-induced precise gene editing in mammalian cells. Nat. Biotechnol. 33 (5), 543–548. 10.1038/nbt.3198 25803306

[B23] Chuang C.-K. Chen C.-H. Huang C.-L. Su Y.-H. Peng S.-H. Lin T.-Y. (2017). Generation of GGTA1 mutant pigs by direct pronuclear microinjection of CRISPR/Cas9 plasmid vectors. Anim. Biotechnol. 28 (3), 174–181. 10.1080/10495398.2016.1246453 27834588

[B24] Cicek Y. A. Luther D. C. Kretzmann J. A. Rotello V. M. (2019). Advances in CRISPR/Cas9 technology for *in vivo* translation. Biol. Pharm. Bull. 42 (3), 304–311. 10.1248/bpb.b18-00811 30828060

[B25] Cofsky J. C. Soczek K. M. Knott G. J. Nogales E. Doudna J. A. (2022). CRISPR–Cas9 bends and twists DNA to read its sequence. Nat. Struct. Mol. Biol. 29 (4), 395–402. 10.1038/s41594-022-00756-0 35422516 PMC9189902

[B26] Cornu T. I. Mussolino C. Cathomen T. (2017). Refining strategies to translate genome editing to the clinic. Nat. Med. 23 (4), 415–423. 10.1038/nm.4313 28388605

[B27] Crispo M. Mulet A. Tesson L. Barrera N. Cuadro F. dos Santos-Neto P. (2015). Efficient generation of myostatin knock-out sheep using CRISPR/Cas9 technology and microinjection into zygotes. PloS one 10 (8), e0136690. 10.1371/journal.pone.0136690 26305800 PMC4549068

[B28] Crudele J. M. Chamberlain J. S. (2018). Cas9 immunity creates challenges for CRISPR gene editing therapies. Nat. Commun. 9 (1), 3497. 10.1038/s41467-018-05843-9 30158648 PMC6115392

[B29] Cullot G. Boutin J. Toutain J. Prat F. Pennamen P. Rooryck C. (2019). CRISPR-Cas9 genome editing induces megabase-scale chromosomal truncations. Nat. Commun. 10 (1), 1136. 10.1038/s41467-019-09006-2 30850590 PMC6408493

[B30] Deng Y. Kizer M. Rada M. Sage J. Wang X. Cheon D.-J. (2018). Intracellular delivery of nanomaterials via an inertial microfluidic cell hydroporator. Nano Lett. 18 (4), 2705–2710. 10.1021/acs.nanolett.8b00704 29569926

[B31] Ding X. Stewart M. Sharei A. Weaver J. C. Langer R. S. Jensen K. F. (2017). High-throughput nuclear delivery and rapid expression of DNA via mechanical and electrical cell-membrane disruption. Nat. Biomed. Eng. 1, 0039. 10.1038/s41551-017-0039 28932622 PMC5602535

[B32] DiTommaso T. Cole J. M. Cassereau L. Buggé J. A. Hanson J. L. S. Bridgen D. T. (2018). Cell engineering with microfluidic squeezing preserves functionality of primary immune cells *in vivo* . Proc. Natl. Acad. Sci. U. S. A. 115(46), E10907–E10914. 10.1073/pnas.1809671115 30381459 PMC6243275

[B33] Dixit H. G. Starr R. Dundon M. L. Pairs P. I. Yang X. Zhang Y. (2020). Massively-parallelized, deterministic mechanoporation for intracellular delivery. Nano Lett. 20 (2), 860–867. 10.1021/acs.nanolett.9b03175 31647675 PMC8210888

[B34] Eusébio D. Almeida A. M. Alves J. M. Maia C. J. Queiroz J. A. Sousa F. (2021). The performance of minicircle DNA versus parental plasmid in p53 gene delivery into HPV-18-Infected cervical cancer cells. Nucleic Acid. Ther. 31 (1), 82–91. 10.1089/nat.2020.0904 33252302

[B35] Finn J. D. Smith A. R. Patel M. C. Shaw L. Youniss M. R. van Heteren J. (2018). A single administration of CRISPR/Cas9 lipid nanoparticles achieves robust and persistent *in vivo* genome editing. Cell Rep. 22 (9), 2227–2235. 10.1016/j.celrep.2018.02.014 29490262

[B36] Florian M. Wang J. P. Deng Y. Souza-Moreira L. Stewart D. J. Mei S. H. J. (2021). Gene engineered mesenchymal stem cells: Greater transgene expression and efficacy with minicircle vs. plasmid DNA vectors in a mouse model of acute lung injury. Stem Cell Res. Ther. 12 (1), 184. 10.1186/s13287-021-02245-5 33726829 PMC7962085

[B37] Francis C. Wroblewska L. Pegman P. Amiji M. (2022). Systemic biodistribution and hepatocyte-specific gene editing with CRISPR/Cas9 using hyaluronic acid-based nanoparticles. Nanomedicine Nanotechnol. Biol. Med. 40, 102488. 10.1016/j.nano.2021.102488 34748964

[B38] Frangoul H. Altshuler D. Cappellini M. D. Chen Y.-S. Domm J. Eustace B. K. (2021). CRISPR-Cas9 gene editing for sickle cell disease and β-thalassemia. N. Engl. J. Med. Overseas. Ed. 384 (3), 252–260. 10.1056/nejmoa2031054 33283989

[B39] Gaudelli N. M. Komor A. C. Rees H. A. Packer M. S. Badran A. H. Bryson D. I. (2017). Programmable base editing of A·T to G·C in genomic DNA without DNA cleavage. Nature 551 (7681), 464–471. 10.1038/nature24644 29160308 PMC5726555

[B40] Gehl J. (2003). Electroporation: Theory and methods, perspectives for drug delivery, gene therapy and research. Acta Physiol. Scand. 177 (4), 437–447. 10.1046/j.1365-201X.2003.01093.x 12648161

[B41] Gillmore J. D. Gane E. Taubel J. Kao J. Fontana M. Maitland M. L. (2021). CRISPR-Cas9 *in vivo* gene editing for transthyretin amyloidosis. N. Engl. J. Med. Overseas. Ed. 385 (6), 493–502. 10.1056/nejmoa2107454 34215024

[B42] Guo J. Wan T. Li B. Pan Q. Xin H. Qiu Y. (2021). Rational design of poly (disulfide) s as a universal platform for delivery of CRISPR-Cas9 machineries toward therapeutic genome editing. ACS Cent. Sci. 7 (6), 990–1000. 10.1021/acscentsci.0c01648 34235260 PMC8227594

[B161] Han X. Liu Z. Jo M. C. Zhang K. Li Y. (2015). CRISPR-Cas9 delivery to hard-to-transfect cells via membrane deformation. Sci. Adv. 1 (7), e1500454. 10.1126/sciadv.1500454 26601238 PMC4643799

[B162] Han X. Liu Z. Ma Y. Zhang K. Qin L. (2017). Cas9 Ribonucleoprotein Delivery via Microfluidic Cell-Deformation Chip for Human T-Cell Genome Editing and Immunotherapy. Adv. Biosyst. 1 (1–2), 1600007. 10.1002/adbi.201600007 32646183

[B43] Hashimoto M. Takemoto T. (2015). Electroporation enables the efficient mRNA delivery into the mouse zygotes and facilitates CRISPR/Cas9-based genome editing. Sci. Rep. 5 (1), 11315–11318. 10.1038/srep11315 26066060 PMC4463957

[B44] Hassett K. J. Benenato K. E. Jacquinet E. Lee A. Woods A. Yuzhakov O. (2019). Optimization of lipid nanoparticles for intramuscular administration of mRNA vaccines. Mol. Ther. - Nucleic Acids 15, 1–11. 10.1016/j.omtn.2019.01.013 30785039 PMC6383180

[B45] Helfield B. Chen X. Watkins S. C. Villanueva F. S. (2016). Biophysical insight into mechanisms of sonoporation. Proc. Natl. Acad. Sci. U. S. A. 113 (36), 9983–9988. 10.1073/pnas.1606915113 27551081 PMC5018802

[B46] Hendel A. Bak R. O. Clark J. T. Kennedy A. B. Ryan D. E. Roy S. (2015). Chemically modified guide RNAs enhance CRISPR-Cas genome editing in human primary cells. Nat. Biotechnol. 33 (9), 985–989. 10.1038/nbt.3290 26121415 PMC4729442

[B47] Hirakawa Matthew P. Krishnakumar R. Timlin J. A. Carney J. P. Butler K. S. (2020). Gene editing and CRISPR in the clinic: Current and future perspectives. Biosci. Rep. 40 (4), BSR20200127. 10.1042/bsr20200127 32207531 PMC7146048

[B48] Hirotsune S. Kiyonari H. Jin M. Kumamoto K. Yoshida K. Shinohara M. (2020). Enhanced homologous recombination by the modulation of targeting vector ends. Sci. Rep. 10 (1), 2518. 10.1038/s41598-020-58893-9 32054870 PMC7018964

[B49] Hou X. Zaks T. Langer R. Dong Y. (2021). Lipid nanoparticles for mRNA delivery. Nat. Rev. Mat. 6 (12), 1078–1094. 10.1038/s41578-021-00358-0 PMC8353930 34394960

[B50] Hruscha A. Schmid B. (2015). “Generation of zebrafish models by CRISPR/Cas9 genome editing,” in Neuronal cell death (Springer), 341–350. 10.1007/978-1-4939-2152-2_24 25431076

[B51] Hu Z. Wang Y. Liu Q. Qiu Y. Zhong Z. Li K. (2021). Improving the precision of base editing by bubble hairpin single guide RNA. mBio 12 (2), e00342-21. 10.1128/mBio.00342-21 33879582 PMC8092237

[B52] Hur J. Park I. Lim K. M. Doh J. Cho S.-G. Chung A. J. (2020). Microfluidic cell stretching for highly effective gene delivery into hard-to-transfect primary cells. ACS Nano 14 (11), 15094–15106. 10.1021/acsnano.0c05169 33034446

[B53] Janjua T. I. Cao Y. Yu C. Popat A. (2021). Clinical translation of silica nanoparticles. Nat. Rev. Mat. 6 (12), 1072–1074. 10.1038/s41578-021-00385-x PMC8496429 34642607

[B54] Jayaraman M. Ansell S. M. Mui B. L. Tam Y. K. Chen J. Du X. (2012). Maximizing the potency of siRNA lipid nanoparticles for hepatic gene silencing *in vivo* . Angew. Chem. Int. Ed. Engl. 124 (34), 8657–8661. 10.1002/ange.201203263 PMC3470698 22782619

[B55] Jinek M. Chylinski K. Fonfara I. Hauer M. Doudna J. A. Charpentier E. (2012). A programmable dual-RNA–guided DNA endonuclease in adaptive bacterial immunity. science 337 (6096), 816–821. 10.1126/science.1225829 22745249 PMC6286148

[B56] Jinek M. East A. Cheng A. Lin S. Ma E. Doudna J. (2013). RNA-programmed genome editing in human cells. Elife 2, e00471. 10.7554/eLife.00471 23386978 PMC3557905

[B57] Jing R. Jiao P. Chen J. Meng X. Wu X. Duan Y. (2021). Cas9-Cleavage sequences in size-reduced plasmids enhance nonviral genome targeting of CARs in primary human T cells. Small Methods 5 (7), e2100071. 10.1002/smtd.202100071 34927998

[B58] Joo B. Hur J. Kim G.-B. Yun S. G. Chung A. J. (2021). Highly efficient transfection of human primary T lymphocytes using droplet-enabled mechanoporation. ACS Nano 15 (8), 12888–12898. 10.1021/acsnano.0c10473 34142817

[B59] Kang G. Carlson D. W. Kang T. H. Lee S. Haward S. J. Choi I. (2020). Intracellular nanomaterial delivery via spiral hydroporation. ACS Nano 14 (3), 3048–3058. 10.1021/acsnano.9b07930 32069037

[B60] Kariko K. Muramatsu H. Welsh F. A. Ludwig J. Kato H. Akira S. (2008). Incorporation of pseudouridine into mRNA yields superior nonimmunogenic vector with increased translational capacity and biological stability. Mol. Ther. 16 (11), 1833–1840. 10.1038/mt.2008.200 18797453 PMC2775451

[B61] Kato-Inui T. Takahashi G. Hsu S. Miyaoka Y. (2018). Clustered regularly interspaced short palindromic repeats (CRISPR)/CRISPR-associated protein 9 with improved proof-reading enhances homology-directed repair. Nucleic acids Res. 46 (9), 4677–4688. 10.1093/nar/gky264 29672770 PMC5961419

[B62] Kelly J. J. Saee-Marand M. Nyström N. N. Evans M. M. Chen Y. Martinez F. M. (2021). Safe harbor-targeted CRISPR-Cas9 homology-independent targeted integration for multimodality reporter gene-based cell tracking. Sci. Adv. 7 (4), eabc3791. 10.1126/sciadv.abc3791 33523917 PMC7817109

[B63] Kenjo E. Hozumi H. Makita Y. Iwabuchi K. A. Fujimoto N. Matsumoto S. (2021). Low immunogenicity of LNP allows repeated administrations of CRISPR-Cas9 mRNA into skeletal muscle in mice. Nat. Commun. 12 (1), 7101. 10.1038/s41467-021-26714-w 34880218 PMC8654819

[B64] Kim S. Kim D. Cho S. W. Kim J. Kim J. S. (2014). Highly efficient RNA-guided genome editing in human cells via delivery of purified Cas9 ribonucleoproteins. Genome Res. 24 (6), 1012–1019. 10.1101/gr.171322.113 24696461 PMC4032847

[B65] Kim S. Koo T. Jee H. G. Cho H. Y. Lee G. Lim D. G. (2018). CRISPR RNAs trigger innate immune responses in human cells. Genome Res. 28, 367–373. 10.1101/gr.231936.117 PMC5848615 29472270

[B66] Knopp Y. Geis F. K. Heckl D. Horn S. Neumann T. Kuehle J. (2018). Transient retrovirus-based CRISPR/Cas9 all-in-one particles for efficient, targeted gene knockout. Mol. Ther. - Nucleic Acids 13, 256–274. 10.1016/j.omtn.2018.09.006 30317165 PMC6187057

[B67] Kocak D. D. Josephs E. A. Bhandarkar V. Adkar S. S. Kwon J. B. Gersbach C. A. (2019). Increasing the specificity of CRISPR systems with engineered RNA secondary structures. Nat. Biotechnol. 37 (6), 657–666. 10.1038/s41587-019-0095-1 30988504 PMC6626619

[B68] Komor A. C. Kim Y. B. Packer M. S. Zuris J. A. Liu D. R. (2016). Programmable editing of a target base in genomic DNA without double-stranded DNA cleavage. Nature 533 (7603), 420–424. 10.1038/nature17946 27096365 PMC4873371

[B69] Kowalski P. S. Rudra A. Miao L. Anderson D. G. (2019). Delivering the messenger: Advances in technologies for therapeutic mRNA delivery. Mol. Ther. 27 (4), 710–728. 10.1016/j.ymthe.2019.02.012 30846391 PMC6453548

[B70] Kuo C. Y. Long J. D. Campo-Fernandez B. de Oliveira S. Cooper A. R. Romero Z. (2018). Site-specific gene editing of human hematopoietic stem cells for X-linked hyper-IgM syndrome. Cell Rep. 23 (9), 2606–2616. 10.1016/j.celrep.2018.04.103 29847792 PMC6181643

[B71] Kurata M. Wolf N. K. Lahr W. S. Weg M. T. Kluesner M. G. Lee S. (2018). Highly multiplexed genome engineering using CRISPR/Cas9 gRNA arrays. PloS one 13 (9), e0198714. 10.1371/journal.pone.0198714 30222773 PMC6141065

[B72] Latorre A. Latorre A. Somoza A. (2016). Modified RNAs in CRISPR/Cas9: An old trick works again. Angew. Chem. Int. Ed. 55 (11), 3548–3550. 10.1002/anie.201512002 26880106

[B73] Le Q. A. Tanihara F. Wittayarat M. Namula Z. Sato Y. Lin Q. (2021). Comparison of the effects of introducing the CRISPR/Cas9 system by microinjection and electroporation into porcine embryos at different stages. BMC Res. Notes 14 (1), 7. 10.1186/s13104-020-05412-8 33407863 PMC7788904

[B74] Lee K. Conboy M. Park H. M. Jiang F. Kim H. J. Dewitt M. A. (2017). Nanoparticle delivery of Cas9 ribonucleoprotein and donor DNA *in vivo* induces homology-directed DNA repair. Nat. Biomed. Eng. 1 (11), 889–901. 10.1038/s41551-017-0137-2 29805845 PMC5968829

[B75] Lesueur L. L. Mir L. M. André F. M. (2016). Overcoming the specific toxicity of large plasmids electrotransfer in primary cells *in vitro* . Mol. Ther. - Nucleic Acids 5, e291. 10.1038/mtna.2016.4 27111417 PMC5014460

[B76] Li L. Hu S. Chen X. (2018a). Non-viral delivery systems for CRISPR/Cas9-based genome editing: Challenges and opportunities. Biomaterials 171, 207–218. 10.1016/j.biomaterials.2018.04.031 29704747 PMC5944364

[B77] Li Y. Yang T. Yu Y. Shi N. Yang L. Glass Z. (2018b). Combinatorial library of chalcogen-containing lipidoids for intracellular delivery of genome-editing proteins. Biomaterials 178, 652–662. 10.1016/j.biomaterials.2018.03.011 29549971

[B78] Li Y. Adur M. K. Wang W. Schultz R. B. Hale B. Wierson W. (2021). Effect of ARTEMIS (DCLRE1C) deficiency and microinjection timing on editing efficiency during somatic cell nuclear transfer and *in vitro* fertilization using the CRISPR/Cas9 system. Theriogenology 170, 107–116. 10.1016/j.theriogenology.2021.04.003 34004455 PMC8243557

[B79] Li C. Yang T. Weng Y. Zhang M. Zhao D. Guo S. (2022). Ionizable lipid-assisted efficient hepatic delivery of gene editing elements for oncotherapy. Bioact. Mater. 9, 590–601. 10.1016/j.bioactmat.2021.05.051 34853819 PMC8604671

[B80] Liang X. Potter J. Kumar S. Zou Y. Quintanilla R. Sridharan M. (2015). Rapid and highly efficient mammalian cell engineering via Cas9 protein transfection. J. Biotechnol. 208, 44–53. 10.1016/j.jbiotec.2015.04.024 26003884

[B81] Liang X. Potter J. Kumar S. Ravinder N. Chesnut J. D. (2017). Enhanced CRISPR/Cas9-mediated precise genome editing by improved design and delivery of gRNA, Cas9 nuclease, and donor DNA. J. Biotechnol. 241, 136–146. 10.1016/j.jbiotec.2016.11.011 27845164

[B82] Liang Y. Wu S. Han W. Wang J. Xu C. Shi J. (2022). Visualizing single-nucleotide variations in a nuclear genome using colocalization of dual-engineered CRISPR probes. Anal. Chem. 94, 11745–11752. 10.1021/acs.analchem.2c01208 35975698

[B83] Liu C. Wan T. Wang H. Zhang S. Ping Y. Cheng Y. (2019a). A boronic acid–rich dendrimer with robust and unprecedented efficiency for cytosolic protein delivery and CRISPR-Cas9 gene editing. Sci. Adv. 5 (6), eaaw8922. 10.1126/sciadv.aaw8922 31206027 PMC6561739

[B84] Liu J. Chang J. Jiang Y. Meng X. Sun T. Mao L. (2019b). Fast and efficient CRISPR/Cas9 genome editing *in vivo* enabled by bioreducible lipid and messenger RNA nanoparticles. Adv. Mat. 31 (33), 1902575. 10.1002/adma.201902575 PMC6732788 31215123

[B85] Liu Q. Wang C. Zheng Y. Zhao Y. Wang Y. Hao J. (2020). Virus-like nanoparticle as a co-delivery system to enhance efficacy of CRISPR/Cas9-based cancer immunotherapy. Biomaterials 258, 120275. 10.1016/j.biomaterials.2020.120275 32798741

[B86] Liu S. Cheng Q. Wei T. Yu X. Johnson L. T. Farbiak L. (2021). Membrane-destabilizing ionizable phospholipids for organ-selective mRNA delivery and CRISPR–Cas gene editing. Nat. Mat. 20 (5), 701–710. 10.1038/s41563-020-00886-0 PMC8188687 33542471

[B87] Lomova A. Clark D. N. Campo-Fernandez B. Flores-Bjurström C. Kaufman M. L. Fitz-Gibbon S. (2018). Improving gene editing outcomes in human hematopoietic stem and progenitor cells by temporal control of DNA repair. Stem Cells 37 (2), 284–294. 10.1002/stem.2935 30372555 PMC6368869

[B88] Lou G. Anderluzzi G. Schmidt S. T. Woods S. Gallorini S. Brazzoli M. (2020). Delivery of self-amplifying mRNA vaccines by cationic lipid nanoparticles: The impact of cationic lipid selection. J. Control. Release 325, 370–379. 10.1016/j.jconrel.2020.06.027 32619745

[B89] Ma K. Li W. Zhu G. Sun S. Chi H. Yin Y. (2021). Functionalized PDA/DEX-PEI@ HA nanoparticles combined with sleeping-beauty transposons for multistage targeted delivery of CRISPR/Cas9 gene. Biomed. Pharmacother. 142, 112061. 10.1016/j.biopha.2021.112061 34449313

[B90] Magis W. DeWitt M. A. Wyman S. K. Vu J. T. Heo S.-J. Shao S. J. (2022). High-level correction of the sickle mutation is amplified *in vivo* during erythroid differentiation. iScience 25 (6), 104374. 10.1016/j.isci.2022.104374 35633935 PMC9130532

[B91] Maier M. A. Jayaraman M. Matsuda S. Liu J. Barros S. Querbes W. (2013). Biodegradable lipids enabling rapidly eliminated lipid nanoparticles for systemic delivery of RNAi therapeutics. Mol. Ther. 21 (8), 1570–1578. 10.1038/mt.2013.124 23799535 PMC3734658

[B92] Martin-Martin I. Aryan A. Meneses C. Adelman Z. N. Calvo E. (2018). Optimization of sand fly embryo microinjection for gene editing by CRISPR/Cas9. PLoS Negl. Trop. Dis. 12 (9), e0006769. 10.1371/journal.pntd.0006769 30180160 PMC6150542

[B93] Maruyama T. Dougan S. K. Truttmann M. C. Bilate A. M. Ingram J. R. Ploegh H. L. (2015). Increasing the efficiency of precise genome editing with CRISPR-Cas9 by inhibition of nonhomologous end joining. Nat. Biotechnol. 33 (5), 538–542. 10.1038/nbt.3190 25798939 PMC4618510

[B94] Miller J. B. Siegwart D. J. (2018). Design of synthetic materials for intracellular delivery of RNAs: From siRNA-mediated gene silencing to CRISPR/Cas gene editing. Nano Res. 11 (10), 5310–5337. 10.1007/s12274-018-2099-4

[B95] Moon S. B. Kim D. Y. Ko J.-H. Kim J.-S. Kim Y.-S. (2019). Improving CRISPR genome editing by engineering guide RNAs. Trends Biotechnol. 37 (8), 870–881. 10.1016/j.tibtech.2019.01.009 30846198

[B96] Mout R. Ray M. Yesilbag Tonga G. Lee Y.-W. Tay T. Sasaki K. (2017). Direct cytosolic delivery of CRISPR/Cas9-ribonucleoprotein for efficient gene editing. ACS Nano 11 (3), 2452–2458. 10.1021/acsnano.6b07600 28129503 PMC5848212

[B97] Mullard A. (2019). First *in vivo* CRISPR candidate enters the clinic. Nat. Rev. Drug Discov. 18 (9), 656–657. 10.1038/d41573-019-00140-6 31477868

[B98] Munye M. M. Tagalakis A. D. Barnes J. L. Brown R. E. McAnulty R. J. Howe S. J. (2016). Minicircle DNA provides enhanced and prolonged transgene expression following airway gene transfer. Sci. Rep. 6 (1), 23125. 10.1038/srep23125 26975732 PMC4792149

[B99] Nault J.-C. Datta S. Imbeaud S. Franconi A. Mallet M. Couchy G. (2015). Recurrent AAV2-related insertional mutagenesis in human hepatocellular carcinomas. Nat. Genet. 47 (10), 1187–1193. 10.1038/ng.3389 26301494

[B100] Nguyen D. N. Roth T. L. Li P. J. Chen P. A. Apathy R. Mamedov M. R. (2020). Polymer-stabilized Cas9 nanoparticles and modified repair templates increase genome editing efficiency. Nat. Biotechnol. 38 (1), 44–49. 10.1038/s41587-019-0325-6 31819258 PMC6954310

[B101] Noureddine A. Maestas-Olguin A. Saada E. A. LaBauve A. E. Agola J. O. Baty K. E. (2020). Engineering of monosized lipid-coated mesoporous silica nanoparticles for CRISPR delivery. Acta Biomater. 114, 358–368. 10.1016/j.actbio.2020.07.027 32702530

[B102] Pan Y. Yang J. Luan X. Liu X. Li X. Yang J. (2019). Near-infrared upconversion–activated CRISPR-cas9 system: A remote-controlled gene editing platform. Sci. Adv. 5 (4), eaav7199. 10.1126/sciadv.aav7199 30949579 PMC6447385

[B103] Pandelakis M. Delgado E. Ebrahimkhani M. R. (2020). CRISPR-based synthetic transcription factors *in vivo*: The future of therapeutic cellular programming. Cell Syst. 10 (1), 1–14. 10.1016/j.cels.2019.10.003 31972154 PMC7175797

[B104] Ping Y. Liu C. Zhang Z. Liu K. L. Chen J. Li J. (2011). Chitosan-graft-(PEI-β-cyclodextrin) copolymers and their supramolecular PEGylation for DNA and siRNA delivery. Biomaterials 32 (32), 8328–8341. 10.1016/j.biomaterials.2011.07.038 21840593

[B105] Qin W. Wang H. (2019). “Delivery of CRISPR-Cas9 into mouse zygotes by electroporation,” in Microinjection (Springer), 179–190. 10.1007/978-1-4939-8831-0_10 30353514

[B106] Qiu M. Glass Z. Chen J. Haas M. Jin X. Zhao X. (2021). Lipid nanoparticle-mediated codelivery of Cas9 mRNA and single-guide RNA achieves liver-specific *in vivo* genome editing of Angptl3. Proc. Natl. Acad. Sci. U. S. A. 118 (10), e2020401118. 10.1073/pnas.2020401118 33649229 PMC7958351

[B107] Ramesan S. Rezk A. R. Dekiwadia C. Cortez-Jugo C. Yeo L. Y. (2018). Acoustically-mediated intracellular delivery. Nanoscale 10 (27), 13165–13178. 10.1039/c8nr02898b 29964280

[B108] Raveux A. Vandormael-Pournin S. Cohen-Tannoudji M. (2017). Optimization of the production of knock-in alleles by CRISPR/Cas9 microinjection into the mouse zygote. Sci. Rep. 7 (1), 42661. 10.1038/srep42661 28209967 PMC5314402

[B109] Ray M. Lee Y.-W. Hardie J. Mout R. Yeşilbag Tonga G. Farkas M. E. (2018). CRISPRed macrophages for cell-based cancer immunotherapy. Bioconjug. Chem. 29 (2), 445–450. 10.1021/acs.bioconjchem.7b00768 29298051 PMC6063311

[B110] Redding S. Sternberg S. H. Marshall M. Gibb B. Bhat P. Guegler C. K. (2015). Surveillance and processing of foreign DNA by the *Escherichia coli* CRISPR-Cas system. Cell 163 (4), 854–865. 10.1016/j.cell.2015.10.003 26522594 PMC4636941

[B111] Rich J. Tian Z. Huang T. J. (2022). Sonoporation: Past, present, and future. Adv. Mat. Technol. 7 (1), 2100885. 10.1002/admt.202100885 PMC8992730 35399914

[B112] Roth T. L. Puig-Saus C. Yu R. Shifrut E. Carnevale J. Li P. J. (2018). Reprogramming human T cell function and specificity with non-viral genome targeting. Nature 559 (7714), 405–409. 10.1038/s41586-018-0326-5 29995861 PMC6239417

[B113] Rui Y. Varanasi M. Mendes S. Yamagata H. M. Wilson D. R. Green J. J. (2020). Poly(Beta-Amino ester) nanoparticles enable nonviral delivery of CRISPR-cas9 plasmids for gene knockout and gene deletion. Mol. Ther. - Nucleic Acids 20, 661–672. 10.1016/j.omtn.2020.04.005 32380416 PMC7210380

[B114] Rui Y. Wilson D. R. Choi J. Varanasi M. Sanders K. Karlsson J. (2019a). Carboxylated branched poly (β-amino ester) nanoparticles enable robust cytosolic protein delivery and CRISPR-Cas9 gene editing. Sci. Adv. 5 (12), eaay3255. 10.1126/sciadv.aay3255 31840076 PMC6897553

[B115] Rui Y. Wilson D. R. Sanders K. Green J. J. (2019b). Reducible branched ester-amine quadpolymers (rBEAQs) codelivering plasmid DNA and RNA oligonucleotides enable CRISPR/Cas9 genome editing. ACS Appl. Mat. Interfaces 11 (11), 10472–10480. 10.1021/acsami.8b20206 PMC7309334 30794383

[B116] Ryan D. E. Diamant-Levi T. Steinfeld I. Taussig D. Visal-Shah S. Thakker S. (2022). Phosphonoacetate modifications enhance the stability and editing yields of guide RNAs for Cas9 editors. Biochemistry 56 (30), 3863–3873. 10.1021/acs.biochem.1c00768 PMC10734248 35436085

[B117] Saung M. T. Sharei A. Adalsteinsson V. A. Cho N. Kamath T. Ruiz C. (2016). A size‐selective intracellular delivery platform. Small 12 (42), 5873–5881. 10.1002/smll.201601155 27594517 PMC5337179

[B118] Shahbazi R. Sghia-Hughes G. Reid J. L. Kubek S. Haworth K. G. Humbert O. (2019). Targeted homology-directed repair in blood stem and progenitor cells with CRISPR nanoformulations. Nat. Mat. 18 (10), 1124–1132. 10.1038/s41563-019-0385-5 PMC6754292 31133730

[B119] Sharei A. Zoldan J. Adamo A. Sim W. Y. Cho N. Jackson E. (2013). A vector-free microfluidic platform for intracellular delivery. Proc. Natl. Acad. Sci. U. S. A. 110 (6), 2082–2087. 10.1073/pnas.1218705110 23341631 PMC3568376

[B120] Sharei A. Poceviciute R. Jackson E. L. Cho N. Mao S. Hartoularos G. C. (2014). Plasma membrane recovery kinetics of a microfluidic intracellular delivery platform. Integr. Biol. 6 (4), 470–475. 10.1039/c3ib40215k PMC3966949 24496115

[B121] Sharma G. Sharma A. R. Bhattacharya M. Lee S.-S. Chakraborty C. (2021). CRISPR-Cas9: A preclinical and clinical perspective for the treatment of human diseases. Mol. Ther. 29 (2), 571–586. 10.1016/j.ymthe.2020.09.028 33238136 PMC7854284

[B122] Singh P. Pandit S. Mokkapati V. R. S. S. Garg A. Ravikumar V. Mijakovic I. (2018). Gold nanoparticles in diagnostics and therapeutics for human cancer. Int. J. Mol. Sci. 19 (7), 1979. 10.3390/ijms19071979 PMC6073740 29986450

[B123] Stadtmauer E. A. Fraietta J. A. Davis M. M. Cohen A. D. Weber K. L. Lancaster E. (2020). CRISPR-engineered T cells in patients with refractory cancer. Science 367 (6481), eaba7365. 10.1126/science.aba7365 32029687 PMC11249135

[B124] Štimac A. Šekutor M. Mlinarić-Majerski K. Frkanec L. Frkanec R. (2017). Adamantane in drug delivery systems and surface recognition. Molecules 22 (2), 297. 10.3390/molecules22020297 PMC6155684 28212339

[B125] Svoboda P. Di Cara A. (2006). Hairpin RNA: A secondary structure of primary importance. Cell. Mol. Life Sci. 63 (7-8), 901–908. 10.1007/s00018-005-5558-5 16568238 PMC11136179

[B126] Tang Q. Liu J. Jiang Y. Zhang M. Mao L. Wang M. (2019). Cell-selective messenger RNA delivery and CRISPR/Cas9 genome editing by modulating the interface of phenylboronic acid-derived lipid nanoparticles and cellular surface sialic acid. ACS Appl. Mat. Interfaces 11 (50), 46585–46590. 10.1021/acsami.9b17749 31763806

[B127] Tang H. Zhao X. Jiang X. (2021). Synthetic multi-layer nanoparticles for CRISPR-Cas9 genome editing. Adv. Drug Deliv. Rev. 168, 55–78. 10.1016/j.addr.2020.03.001 32147450

[B128] Tay A. Melosh N. (2021). Mechanical stimulation after centrifuge-free nano-electroporative transfection is efficient and maintains long-term T cell functionalities. Small 17 (38), e2103198. 10.1002/smll.202103198 34396686 PMC8475193

[B129] Tu J. Yu A. C. H. (2022). Ultrasound-mediated drug delivery: Sonoporation mechanisms, biophysics, and critical factors. BME Front. 2022, 1–17. 10.34133/2022/9807347 37850169 PMC10521752

[B130] Uvizl A. Goswami R. Gandhi S. D. Augsburg M. Buchholz F. Guck J. (2021). Efficient and gentle delivery of molecules into cells with different elasticity via Progressive Mechanoporation. Lab. Chip 21 (12), 2437–2452. 10.1039/D0LC01224F 33977944 PMC8204113

[B131] Vaidyanathan S. Azizian K. T. Haque A. Henderson J. M. Hendel A. Shore S. (2018). Uridine depletion and chemical modification increase Cas9 mRNA activity and reduce immunogenicity without HPLC purification. Mol. Ther. - Nucleic Acids 12, 530–542. 10.1016/j.omtn.2018.06.010 30195789 PMC6076213

[B132] Wagner T. E. Becraft J. R. Bodner K. Teague B. Zhang X. Woo A. (2018). Small-molecule-based regulation of RNA-delivered circuits in mammalian cells. Nat. Chem. Biol. 14 (11), 1043–1050. 10.1038/s41589-018-0146-9 30327560

[B133] Walther J. Wilbie D. Tissingh V. S. Öktem M. van der Veen H. Lou B. (2022). Impact of formulation conditions on lipid nanoparticle characteristics and functional delivery of CRISPR RNP for gene knock-out and correction. Pharmaceutics 14 (1), 213. 10.3390/pharmaceutics14010213 35057110 PMC8778360

[B134] Wan T. Chen Y. Pan Q. Xu X. Kang Y. Gao X. (2020). Genome editing of mutant KRAS through supramolecular polymer-mediated delivery of Cas9 ribonucleoprotein for colorectal cancer therapy. J. Control. Release 322, 236–247. 10.1016/j.jconrel.2020.03.015 32169537

[B135] Wang M. Zuris J. A. Meng F. Rees H. Sun S. Deng P. (2016). Efficient delivery of genome-editing proteins using bioreducible lipid nanoparticles. Proc. Natl. Acad. Sci. U. S. A. 113 (11), 2868–2873. 10.1073/pnas.1520244113 26929348 PMC4801296

[B136] Wang H. X. Song Z. Lao Y. H. Xu X. Gong J. Cheng D. (2018a). Nonviral gene editing via CRISPR/Cas9 delivery by membrane-disruptive and endosomolytic helical polypeptide. Proc. Natl. Acad. Sci. U. S. A. 115 (19), 4903–4908. 10.1073/pnas.1712963115 29686087 PMC5948953

[B137] Wang P. Zhang L. Zheng W. Cong L. Guo Z. Xie Y. (2018b). Thermo‐triggered release of CRISPR‐Cas9 system by lipid‐encapsulated gold nanoparticles for tumor therapy. Angew. Chem. Int. Ed. 57 (6), 1491–1496. 10.1002/anie.201708689 29282854

[B138] Wang Y. Shahi P. K. Wang X. Xie R. Zhao Y. Wu M. (2021). *In vivo* targeted delivery of nucleic acids and CRISPR genome editors enabled by GSH-responsive silica nanoparticles. J. Control. Release 336, 296–309. 10.1016/j.jconrel.2021.06.030 34174352 PMC8383466

[B139] Wei T. Cheng Q. Min Y.-L. Olson E. N. Siegwart D. J. (2020). Systemic nanoparticle delivery of CRISPR-Cas9 ribonucleoproteins for effective tissue specific genome editing. Nat. Commun. 11 (1), 3232. 10.1038/s41467-020-17029-3 32591530 PMC7320157

[B140] Wu Z. Yang H. Colosi P. (2010). Effect of genome size on AAV vector packaging. Mol. Ther. 18 (1), 80–86. 10.1038/mt.2009.255 19904234 PMC2839202

[B141] Wu Y. Zhou H. Fan X. Zhang Y. Zhang M. Wang Y. (2015). Correction of a genetic disease by CRISPR-Cas9-mediated gene editing in mouse spermatogonial stem cells. Cell Res. 25 (1), 67–79. 10.1038/cr.2014.160 25475058 PMC4650588

[B142] Wu W. Lu Z. Li F. Wang W. Qian N. Duan J. (2017). Efficient *in vivo* gene editing using ribonucleoproteins in skin stem cells of recessive dystrophic epidermolysis bullosa mouse model. Proc. Natl. Acad. Sci. U. S. A. 114(7), 1660–1665. 10.1073/pnas.1614775114 28137859 PMC5321012

[B143] Wu M. Chen K. Yang S. Wang Z. Huang P.-H. Mai J. (2018). High-throughput cell focusing and separation via acoustofluidic tweezers. Lab. Chip 18 (19), 3003–3010. 10.1039/C8LC00434J 30131991 PMC6203445

[B144] Xiong R. Raemdonck K. Peynshaert K. Lentacker I. De Cock I. Demeester J. (2014). Comparison of gold nanoparticle mediated photoporation: Vapor nanobubbles outperform direct heating for delivering macromolecules in live cells. ACS Nano 8 (6), 6288–6296. 10.1021/nn5017742 24870061

[B145] Xu X. Gao D. Wang P. Chen J. Ruan J. Xu J. (2018). Efficient homology-directed gene editing by CRISPR/Cas9 in human stem and primary cells using tube electroporation. Sci. Rep. 8 (1), 11649. 10.1038/s41598-018-30227-w 30076383 PMC6076306

[B146] Xu X. Koivisto O. Liu C. Zhou J. Miihkinen M. Jacquemet G. (2021). Effective delivery of the CRISPR/Cas9 system enabled by functionalized mesoporous silica nanoparticles for GFP‐tagged paxillin knock‐in. Adv. Ther. (Weinh). 4 (1), 2000072. 10.1002/adtp.202000072

[B147] Yamagishi A. Matsumoto D. Kato Y. Honda Y. Morikawa M. Iwata F. (2019). Direct delivery of cas9-sgRNA ribonucleoproteins into cells using a nanoneedle array. Appl. Sci. 9 (5), 965. 10.3390/app9050965

[B148] Yang H. Wu J.-J. Tang T. Liu K.-D. Dai C. (2017). CRISPR/Cas9-mediated genome editing efficiently creates specific mutations at multiple loci using one sgRNA in Brassica napus. Sci. Rep. 7 (1), 7489. 10.1038/s41598-017-07871-9 28790350 PMC5548805

[B149] Yang R. Lemaître V. Huang C. Haddadi A. McNaughton R. Espinosa H. D. (2018). Monoclonal cell line generation and CRISPR/Cas9 manipulation via single‐cell electroporation. Small 14 (12), 1702495. 10.1002/smll.201702495 PMC6016377 29430869

[B150] Yang P. Chou S.-J. Li J. Hui W. Liu W. Sun N. (2020). Supramolecular nanosubstrate–mediated delivery system enables CRISPR-Cas9 knockin of hemoglobin beta gene for hemoglobinopathies. Sci. Adv. 6 (43), eabb7107. 10.1126/sciadv.abb7107 33097539 PMC7608838

[B151] Yip B. H. (2020). Recent advances in CRISPR/Cas9 delivery strategies. Biomolecules 10 (6), 839. 10.3390/biom10060839 PMC7356196 32486234

[B152] Yoon S. Wang P. Peng Q. Wang Y. Shung K. K. (2017). Acoustic-transfection for genomic manipulation of single-cells using high frequency ultrasound. Sci. Rep. 7 (1), 5275. 10.1038/s41598-017-05722-1 28706248 PMC5509725

[B153] Zamolo S. J. Darbre T. Reymond J. L. (2020). Transfecting tissue models with CRISPR/Cas9 plasmid DNA using peptide dendrimers. Chem. Commun. 56 (80), 11981–11984. 10.1039/d0cc04750c 32895670

[B154] Zangi L. Lui K. O. von Gise A. Ma Q. Ebina W. Ptaszek L. M. (2013). Modified mRNA directs the fate of heart progenitor cells and induces vascular regeneration after myocardial infarction. Nat. Biotechnol. 31 (10), 898–907. 10.1038/nbt.2682 24013197 PMC4058317

[B155] Zhang L. Wang P. Feng Q. Wang N. Chen Z. Huang Y. (2017a). Lipid nanoparticle-mediated efficient delivery of CRISPR/Cas9 for tumor therapy. NPG Asia Mat. 9 (10), e441. 10.1038/am.2017.185

[B156] Zhang Z. Wang Y. Zhang H. Tang Z. Liu W. Lu Y. (2017b). Hypersonic poration: A new versatile cell poration method to enhance cellular uptake using a piezoelectric nano‐electromechanical device. Small 13 (18), 1602962. 10.1002/smll.201602962 28195400

[B157] Zhang B.-C. Luo B.-Y. Zou J.-J. Wu P.-Y. Jiang J.-L. Le J.-Q. (2020). Co-delivery of sorafenib and CRISPR/Cas9 based on targeted core–shell hollow mesoporous organosilica nanoparticles for synergistic HCC therapy. ACS Appl. Mat. Interfaces 12 (51), 57362–57372. 10.1021/acsami.0c17660 33301289

[B158] Zhang Q. Kuang G. He S. Liu S. Lu H. Li X. (2021a). Chain-shattering Pt (IV)-backboned polymeric nanoplatform for efficient CRISPR/Cas9 gene editing to enhance synergistic cancer therapy. Nano Res. 14 (3), 601–610. 10.1007/s12274-020-3066-4

[B159] Zhang S. Shen J. Li D. Cheng Y. (2021b). Strategies in the delivery of Cas9 ribonucleoprotein for CRISPR/Cas9 genome editing. Theranostics 11 (2), 614–648. 10.7150/thno.47007 33391496 PMC7738854

